# Decontamination of ambient RNA in single-cell RNA-seq with DecontX

**DOI:** 10.1186/s13059-020-1950-6

**Published:** 2020-03-05

**Authors:** Shiyi Yang, Sean E. Corbett, Yusuke Koga, Zhe Wang, W Evan Johnson, Masanao Yajima, Joshua D. Campbell

**Affiliations:** 1 Division of Computational Biomedicine, Department of Medicine, Boston University School of Medicine, Boston, MA USA; 2 Department of Mathematics & Statistics, Boston University, Boston, MA USA

**Keywords:** Bayesian mixture model, Decontamination, Single cell, scRNA-seq

## Abstract

Droplet-based microfluidic devices have become widely used to perform single-cell RNA sequencing (scRNA-seq). However, ambient RNA present in the cell suspension can be aberrantly counted along with a cell’s native mRNA and result in cross-contamination of transcripts between different cell populations. DecontX is a novel Bayesian method to estimate and remove contamination in individual cells. DecontX accurately predicts contamination levels in a mouse-human mixture dataset and removes aberrant expression of marker genes in PBMC datasets. We also compare the contamination levels between four different scRNA-seq protocols. Overall, DecontX can be incorporated into scRNA-seq workflows to improve downstream analyses.

## Background

Single-cell RNA sequencing (scRNA-seq) has emerged as a powerful technique to study complex biological systems at single-cell resolution [1]. Droplet-based scRNA-seq platforms have been widely adopted because of their ability to profile a large number of cells at relatively low cost [2]. These devices work by using droplets to partition cells into nanoliter reaction chambers along with beads harboring oligonucleotide primers with unique barcodes. Within each droplet, cells are lysed and the mRNAs will be tagged with the oligonucleortide primers to create barcoded cDNA after reverse transcription [3–5].

**Fig. 1:**
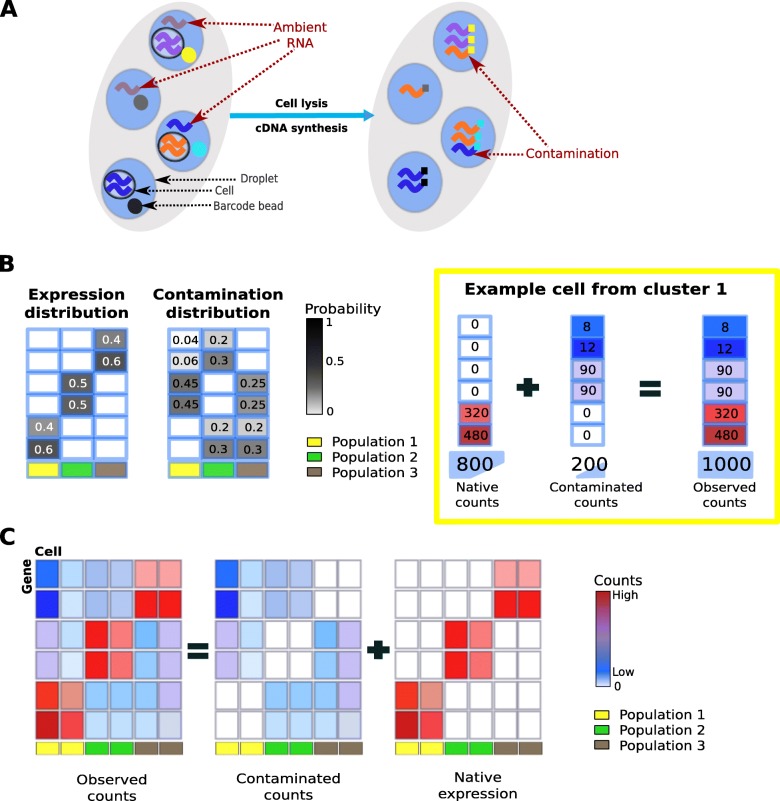
Overview of decontamination with DecontX. **a** In droplet-based microfluidic devices, ambient RNA can be incorporated into droplets along with oligonucleotide-barcoded beads and cells. Both native mRNA from the cell and contaminating ambient RNA will be barcoded and counted within a droplet. **b** Left: DecontX assumes that each cell is a mixture of two multinomial distributions: (1) a distribution of native transcripts from the cell’s true population and (2) a distribution of contaminating transcripts from all other cell populations captured in the assay. Right: simulation of an example cell with 20% contamination. The 800 native transcripts are from the multinomial distribution for cell population 1 while the 200 contaminating transcripts are derived from a probability distribution that is a weighted combination of the 2 other populations. **c** DecontX will take an expression count matrix and cell cluster labels and estimate matrices of native expression and contamination from ambient RNA

Despite their many advantages, droplet-based single-cell technologies can suffer from the presence of cross-contamination from ambient RNA in each droplet. Ambient RNA is the pool of mRNA molecules that have been released in the cell suspension, likely from cells that are stressed or have undergone apoptosis. Cross-contamination occurs when the ambient RNA gets incorporated into the droplets and is barcoded and amplified along with a cell’s native mRNA (Fig. 1a). Contamination from ambient RNA is evident when highly expressed cell type-specific genes are observed at low levels in other cell populations. Different proportions of contamination can be found in different droplets depending on the amount of ambient and native mRNA present. Two major goals of many scRNA-seq studies are to cluster cells into subpopulations and identify unique combinations of marker genes that define each cell population [6]. Ambient RNA can hinder these tasks by causing different cell populations to “blend” together and the expression of true marker genes to be detected across multiple cell populations. Beyond ambient RNA, other technical factors may cause contamination between cells such as evaporation in plate-based protocols [7, 8] or barcode swapping during sequencing [9].

Another common problem in scRNA-seq methods is when more than one cell is captured in a droplet, also known as a “doublet” or “multiplet.” In microfluidic systems, the occurrence of doublets is proportional to the concentration of cells in the suspension and capture rate of the device [10–13]. Several computational methods have been developed to detect doublets for scRNA-seq data. Scrublet [10] and DoubletFinder [11] simulate artificial doublets from the original data coordinates in a reduced-dimensional representation, then create doublet score for each barcode by calculating the similarity of its representation with artificial doublets. Other approaches such as demuxlet [12] and scds [13] model gene expression from the original data, then assign doublet to barcodes that have observed expression from genes that are likely to not occur simultaneously. While it is important to identify doublets in scRNA-seq, these approaches do not address the problem of contamination caused by ambient RNA or other experimental factors.

We developed a computational method called DecontX to estimate and remove ambient RNA for scRNA-seq data. We applied DecontX to three datasets to demonstrate its ability to accurately quantify and remove contamination within each cell from other populations and to improve downstream clustering. Applying DecontX to benchmark datasets containing the same cell lines sequenced across four different scRNA-seq methods, including two plate-based (CEL-seq2 and SORT-seq) and two droplet-based (10X Chromium and Drop-seq), generally showed that 10X Chromium had the lowest levels of contamination while CEL-seq2 had the highest. Highly contaminated cells also showed consistency with doublet predictions by Scrublet and demuxlet suggesting that this approach can also support doublet detection.

## Results

To address the issue of contamination, we developed a novel Bayesian method called DecontX that identifies and removes contamination in individual cells. We assume the observed expression of a cell is a mixture of counts from two multinomial distributions: (1) a distribution of native transcript counts from the cell’s actual population and (2) a distribution of contaminating transcript counts from all other cell populations captured in the assay (online methods, Fig. 1b). The native expression distribution for each cell population is characterized by a multinomial parameter *ϕ*_*k*_, where *ϕ*_*kg*_ is the probability of gene *g* being expressed in population *k*. Likewise, the contamination distribution for each cell population *k* is characterized by a multinomial parameter *η*_*k*_, where *η*_*kg*_ is the probability of gene *g* contaminating population *k*. Each individual cell *j* has a parameter *θ*_*j*_, which follows a beta distribution and represents the proportion of counts derived from the native expression distribution. Each transcript count has a hidden state, *y*_*jt*_, which follows a Bernoulli distribution parameterized by *θ*_*j*_ and denotes the transcript’s membership to the native expression distribution (*y*_*jt*_=1) or contamination distribution (*y*_*jt*_=0). This framework is similar to a discrete Bayesian hierarchical model called latent Dirichlet allocation (LDA) [14] where documents are mixtures of *K* topics and each topic is a mixture of words from a predefined vocabulary. However, rather than having *K* different distributions to model the mixtures of counts from different cell populations within each cell, we explicitly define the contamination distribution to be a weighted combination of all other cell population distributions. We use variational inference [15] to approximate posterior distributions to allow fast and scalable inference in large datasets [16]. Ultimately, DecontX will deconvolute a gene-by-cell count matrix and a vector of cell population labels into a matrix of contamination counts and a matrix of native counts which can be used in downstream analyses (Fig. 1c).

**Fig. 2:**
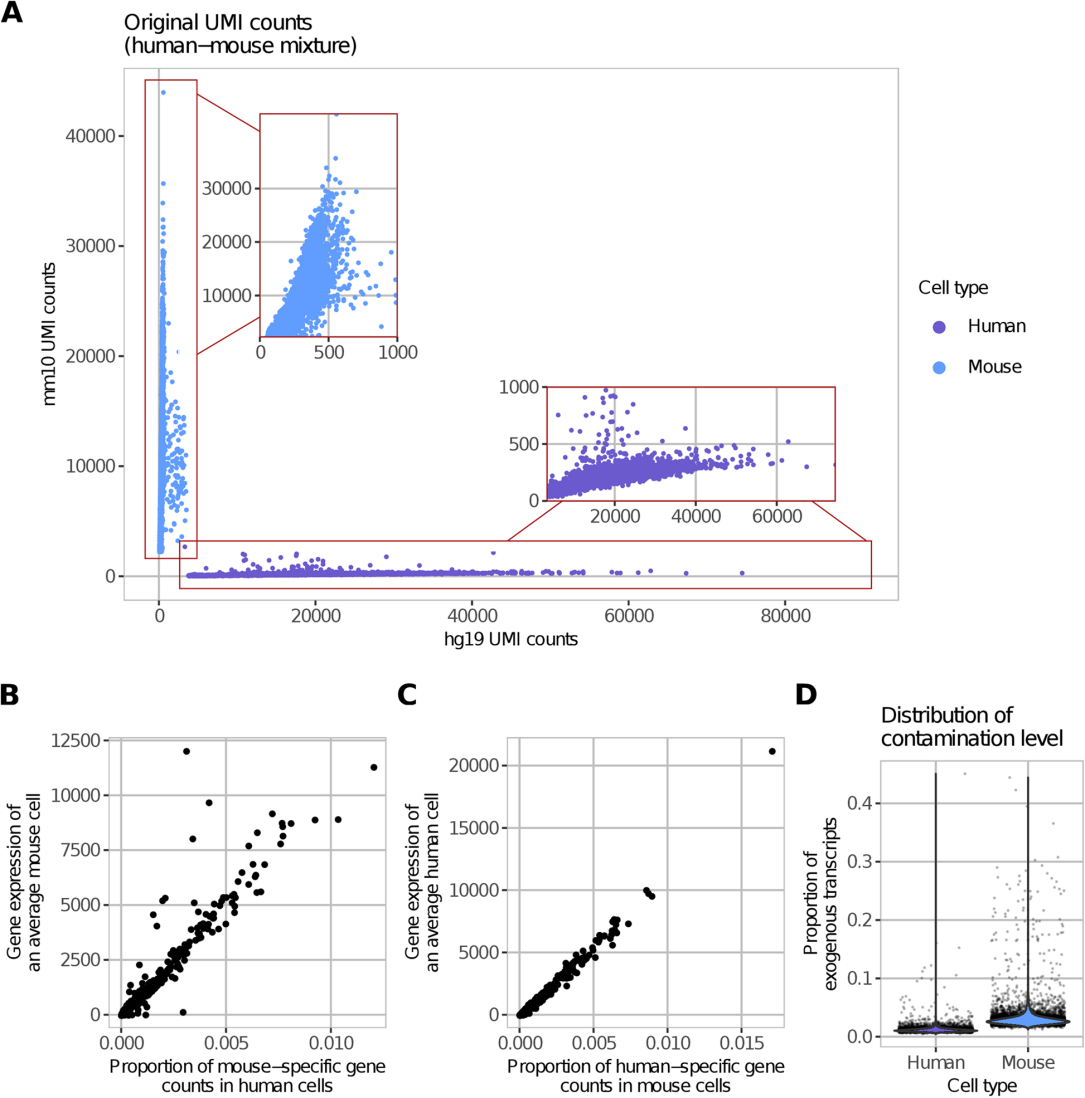
Contamination in a human-mouse cell mixture dataset. **a** The total number of UMIs aligned specifically to the mouse or human genome is plotted for each droplet. **b** The proportion of counts for mouse genes in human cells is highly correlated to the average expression of these genes across all mouse cells indicating that the amount of contamination for each gene is proportional to how highly that gene is expressed in the contaminating cell population. **c** Similarly, the proportion of counts for human genes in the mouse cells is highly correlated to the average expression of those genes across all human cells. **d** While each droplet is predicted to contain a single cell, the median percentage of contamination for human and mouse cells is 1.09% and 2.75%, respectively. The range of contamination is 0.43–45.09% indicating the need for contamination estimation for each individual cell

To demonstrate the accuracy of DecontX, we utilized a public dataset containing a mixture of fresh frozen human embryonic cells (HEK293T) and mouse embryonic fibroblast (NIH3T3) cells from 10X Genomics. Using CellRanger [5], reads were uniquely aligned to a combined human-mouse reference genome (hg19 and mm10) to ensure that only reads specific to each organism will be counted while those that align to the genome of both organisms will be excluded. Cells were classified as human, mouse, or multiplets based on the levels of the organism-specific transcript counts (Additional file 1: Figure S1). The cells predicted to be either mouse or human still exhibited low levels of expression of counts aligning specifically to the other organism (Fig. 2a). The proportion of mouse-specific genes in human cells was highly correlated to the distribution of expression in an average mouse cell (*R* = 0.96; Fig. 2b). Conversely, the proportion of human-specific genes in mouse cells was highly correlated to the distribution of expression in an average human cell (*R* = 0.99; Fig. 2c). These results also show that highly expressed genes in one cell subpopulation are more likely to contribute to contamination in other cell populations. Furthermore, while the median contamination was relatively low (1.09% in human cells and 2.75% in mouse cells), the percentage of contamination varied substantially from cell to cell (0.43–45.09% in human; 1.25–44.43% in mouse; Fig. 2d) and demonstrates the need to have individual estimates of contamination for each cell.

**Fig. 3:**
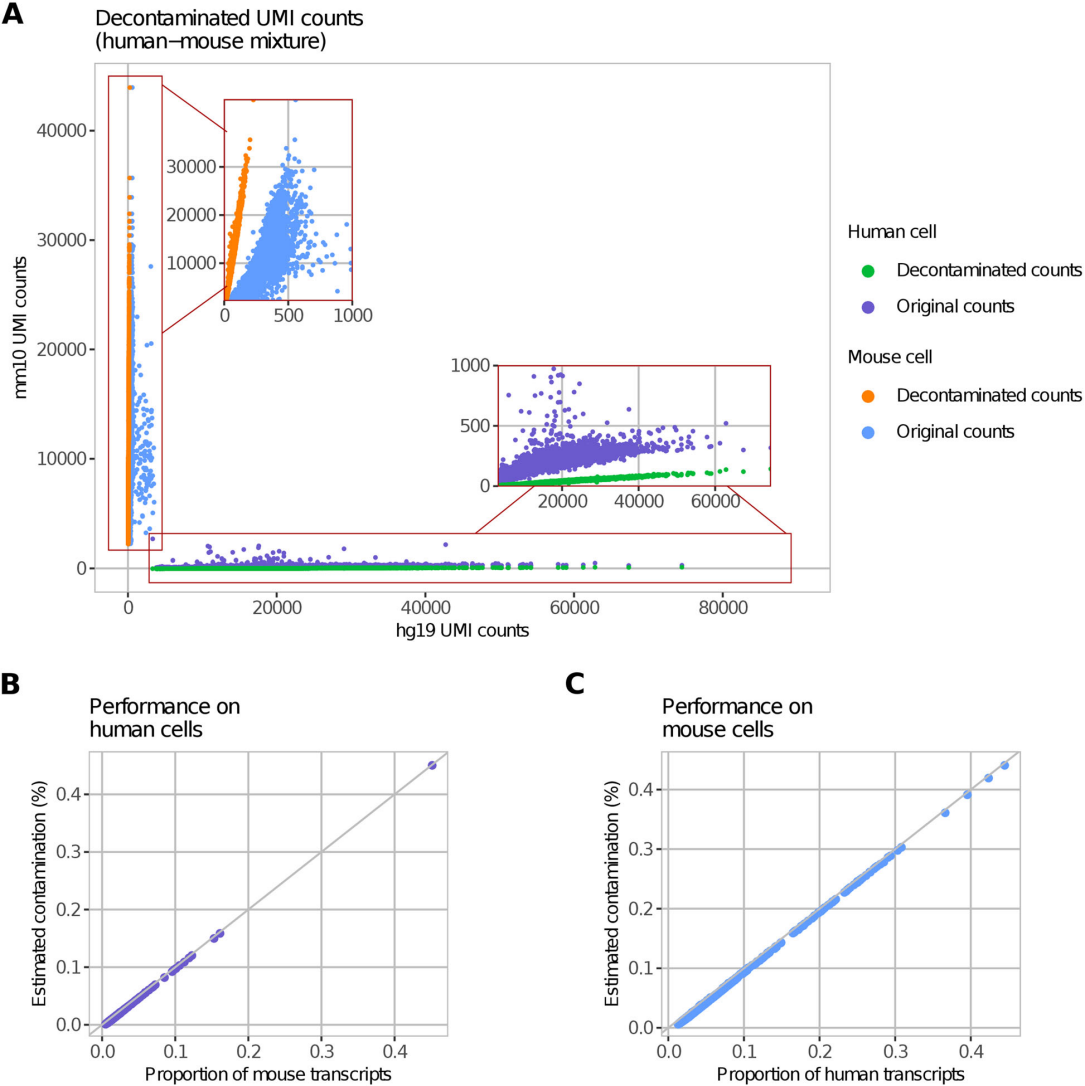
Decontamination of the human-mouse cell mixture dataset. **a** The number of human UMIs is again plotted against the number of mouse UMIs for each droplet before and after decontamination with DecontX. After DecontX, the median percentage of contaminating counts for each droplet is 0.25% (0.12–0.75%). **b**, **c** DecontX-estimated contamination proportion is highly correlated to the known proportion of exogenous transcripts for each droplet predicted to have a human or mouse cell

We applied DecontX to 12,079 non-multiplet cells in the human-mouse mixture dataset. Most of the exogenous transcripts were identified and removed by DecontX (Fig. 3a). The estimated proportion of contamination in individual human cells was highly correlated to the proportion of mouse-specific transcripts in those cells (*R* = 0.99; RMSE = 0.002; Fig. 3b). A high correlation was also observed in mouse cells (*R* = 0.99; RMSE = 0.006; Fig. 3c), demonstrating the ability of DecontX to accurately detect contamination from other cell populations. The estimated gene-level contamination distributions for human or mouse cell populations were also highly correlated to the expression of an average mouse or human cell, respectively (Additional file 1: Figure S2).

**Fig. 4:**
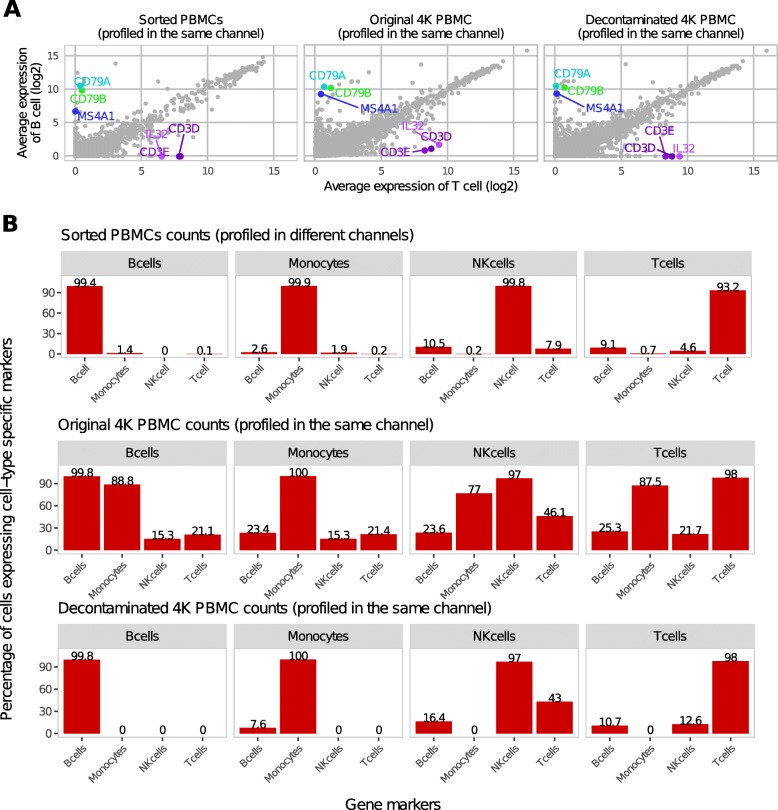
Expression of cell type-specific marker genes before and after decontamination in PBMCs. **a** For each gene, the average expression in the B cell clusters is plotted against the average expression in T cell clusters for three different datasets: data from sorted PMBCs profiled in different channels (left), data from the PBMC 4K before decontamination (middle), and the PBMC 4K data after decontamination with DecontX (right). **b** Percentage of cells expressing specific marker genes for different cell types for three different datasets. Markers included CD79A, CD79B, and MS4A1 for B cells; CD3E and CD3D for T cells; GNLY for NK cells; and LYZ, S100A8, and S100A9 for monocytes

We next sought to understand the effect and extent of contamination in publicly available scRNA-seq datasets of peripheral mononuclear cells (PBMCs). To establish baseline expression of cell type-specific marker genes in a setting with limited possibility for contamination, we examined 4 different immune populations isolated by flow cytometry and profiled with the 10X Genomics Chromium in separate channels [5] (i.e., sorted PBMCs). As each population was isolated and profiled in a different channel, gene markers for a specific immune population were detected at relatively low levels in other populations. For example, the mRNA expression of T cell-specific genes such as CD3E and CD3D were only found 0.07% in the B cells sorted on CD20. Conversely, B cell-specific markers such as CD79A, CD79B, and MS4A1 were only detected in 9.09% of T cells sorted on CD8A or CD4 (Fig. 4a,b). Similarly, low percentages of marker genes of other cell types could be found for B cells and monocytes, monocytes and T cells, T cells and NK cells, NK cells and B cells, and NK cells and monoctyes (Fig. 4b, Additional file 1: Figure S3).

**Fig. 5:**
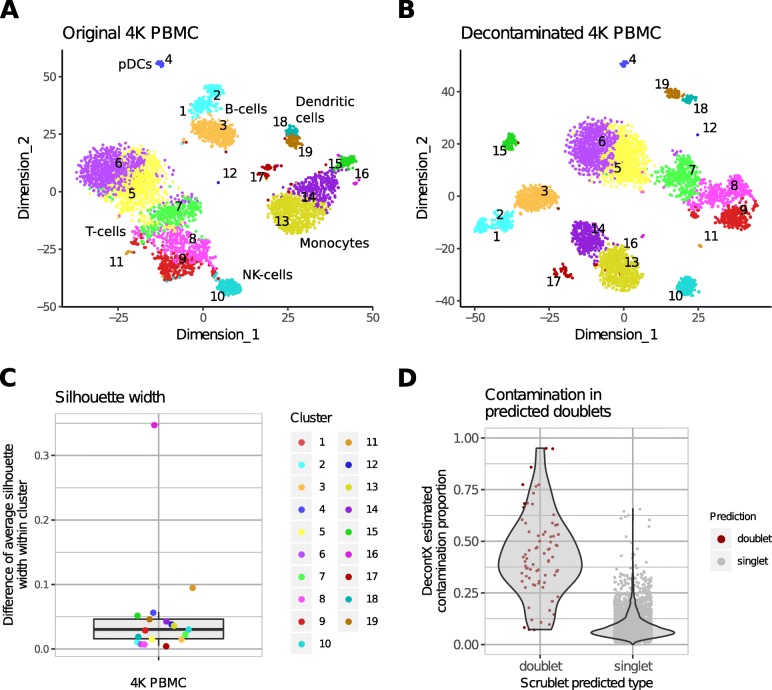
Cluster similarity before and after decontamination. **a** tSNE of 19 cell clusters from the PBMC 4K dataset before decontamination. **b** Decontamination with DecontX improved separation on tSNE between different cell clusters. **c** The mean silhouette width was derived for each cluster before and after decontamination with DecontX. Each point represents the difference in the mean silhouette width for each cluster. All clusters except 17 showed an increase in silhouette width after decontamination. Cluster 17 was predicted to contain mostly doublets by Scrublet. Cluster 1 had only one cell and was not included in the analysis. **d** Predicted doublets had significantly higher levels of estimated contamination compared to singlets. The median contamination for doublets was 41.77% (7.32–95.08%) while the median for singlets was 7.02% (0.07–65.64%)

In the second dataset, over 4000 PBMCs (4K PBMC) were isolated and profiled in a single channel of the 10X Genomics Chromium. Since cluster labels were not available from flow cytometry, we utilized Celda [17] to identify 19 cell populations where each population was a unique combination of 150 gene modules (Additional file 1: Figure S4, S5). In contrast to the previous dataset, higher levels of cell type-specific marker genes could be detected in other cell types including CD3E and CD3D in 21.12% B cell population, and CD79A, CD79B, and MS4A1 in 25.32% T cell population (Fig. 4). Likewise, higher level of a marker gene (GNLY) for NK cells was found in monocytes and B cells, marker genes (LYZ, S100A8, and S100A9) for monocytes in NK cells, B cells, and T cells (Fig. 4b, Additional file 1: Figure S3). We also observed that monocyte marker genes were the most prevalent contamination in other cell types (Fig. 4). Monocytes had the highest median value of total number of UMIs among the four major cell types (Additional file 1: Figure S6A), and the monocyte-specific markers LYZ, S100A8, and S100A9 had higher expression levels than other cell type-specific markers (Additional file 1: Figure S6B). Therefore, monocyte marker genes contributed higher probabilities to the contamination distributions in other cell types compared to marker genes from other cell types. After decontamination, the expression of T cell-specific marker genes was eliminated in B cells and expression of B cell-specific marker genes was eliminated in T cells (Fig. 4). The percentage of cells within each subpopulation that had expression of marker genes from other cell types markedly decreased (Fig. 4b, Additional file 1: Figure S3). The only exception was that 43.03% of NK cells retained some expression of T cell markers. Despite this, overall expression of T cell markers was still significantly reduced in NK cells (*p* value = 0.0005 for CD3D, *p* value = 0.0005 for CD3E; Additional file 1: Figure S3F). Decontaminated counts resulted in improved separation in two dimensions when applying tSNE [18] (Fig. 5a, b). Additionally, the mean silhouette width, a measure of cluster stability and separation, improved from 0.04 on original normalized expression to 0.07 on normalized expression after decontamination (Fig. 5c, Additional file 1: Figure S6C). The highest contamination levels of a cell estimated by DecontX were in cluster 17 (Additional file 1: Figure S6D). Interestingly, cells from cluster 17 were predicted to be doublets by a doublet prediction method Scrublet [10] (Additional file 1: Figure S6D, S6E). Cells predicted to be doublets by Scrublet are associated with higher contamination estimated by DecontX (*p* value < 2e −16, Fig. 5d). Conversely, all cells estimated to have high levels of contamination (>70*%*) were predicted to be doublets by Scrublet suggesting that DecontX contamination estimates can be used as orthogonal information for doublet detection.

**Fig. 6:**
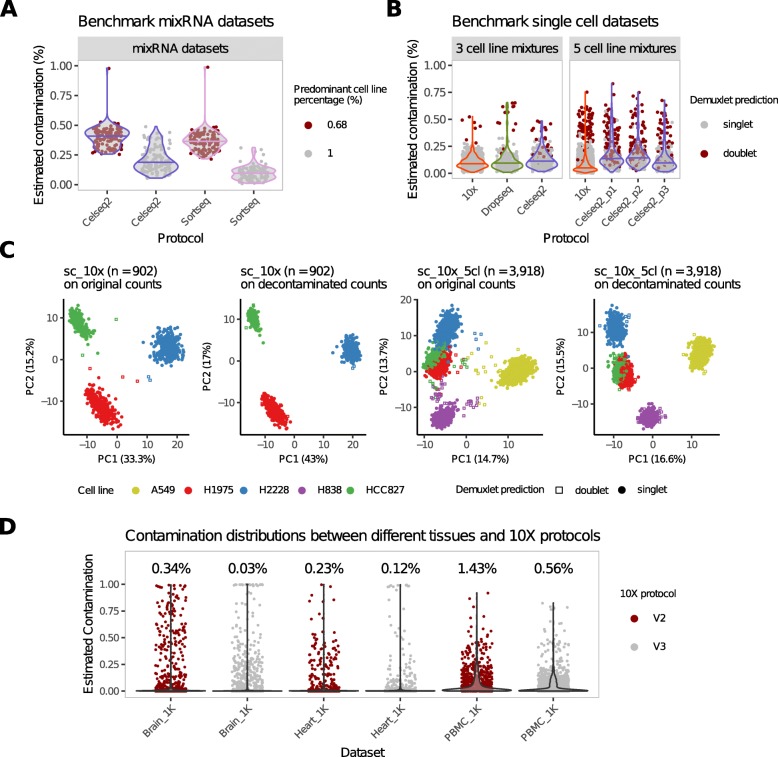
Contamination levels for different scRNA-seq protocols, and contamination levels between different tissues and 10X platforms. **a** Distributions of DecontX-estimated contamination for psudo-cells generated by mixing RNA extracted from three cell lines in ratios of 68%, 16%, and 16% (red), or aliquoting 100% of RNA from one cell line (gray) which were sequenced in either CEL-seq2 or SORT-seq. **b** Distributions of DecontX-estimated contamination for three cell lines that were mixed and sequenced with the 10X Chromium, Drop-seq, or CEL-seq2 protocols. Additionally, five cell lines were mixed and sequenced using CEL-seq2 protocol in three distinct replicates. **c** The three-cell-line (left two columns) mixture dataset and the five-cell-line (right two columns) mixture dataset sequenced with 10X Chromium are shown on two dimensions using PCA with normalized counts with and without decontamination. Decontamination decreased within cluster variability while maintaining the overall relationships between clusters. **d** Distributions of DecontX-estimated contamination for cell types from three different tissues (mouse brain, mouse heart, and PBMC from a healthy donor) profiled using two chemistries (V2 and V3) using 10X Chromium

To examine the degree of contamination produced by different scRNA-seq methods, we applied DecontX to benchmark datasets consisting of human lung adenocarcinoma cell lines profiled with different scRNA-seq methods [7]. The first dataset utilized pseudo-cells generated by mixing RNA extracted from three different cell lines in ratios of 68%, 16%, and 16%. DecontX estimated the median contamination percentages as 41.15% in CEL-seq2 and 36.47% in SORT-seq (Fig. 6a). This is slightly higher than the amount expected from the experimental conditions (32%) suggesting additional cross-contamination occurred in the library preparation process. The possibility of additional contamination is further supported by the fact that the pseudo-cells generated by aliquoting 100% of RNA for one cell line still exhibited a median contamination of 18.08% in CEL-seq2 and 9.09% in SORT-seq (Fig. 6a). Two other datasets consisted of five cell lines (HCC827, H1975, A549, H838, and H2228) mixed in equal proportions and profiled with CEL-seq2 or 10X Chromium as well as three cell lines (HCC827, H1975, and H2228) mixed in equal proportions and profiled with CEL-seq2, Drop-seq with Dolomite equipment, or 10X Chromium. Cell lines profiled with 10X Chromium had the lowest median contamination with 8.81% observed in the three-cell-line data and 4.96% observed in the five-cell-line data. Cells profiled with CEL-seq2 had the highest median contamination with 10.99% observed in three-cell-line data and 13.13%, 13.96%, and 9.22% observed in each of the replicates for the five-cell-line data. The median contamination for Drop-seq (9.29% for the three-cell-line data) is slightly higher than those profiled in 10X Chromium (Fig. 6b). Cells predicted to be doublets were associated with higher contamination estimated by DecontX regardless of protocols used (*p* value < 2e −16). For all protocols examined, decontaminated counts decreased within-cluster variability while preserving relative relationships between clusters in two dimensions from principle component analysis (PCA) (Fig. 6c, Additional file 1: Figure S7).

To investigate the variation of contamination levels between cell types from different tissues and 10X protocols, we applied DecontX to six additional datasets including brain cells and heart cells from an E18 mouse profiled using 10X V2 and V3 chemistries (BrainV2 and BrainV3, HeartV2 and HeartV3) as well as PBMCs from a healthy human donor (PBMCV2 and PBMCV3). All six datasets each had between 712 and 1301 cells detected. The median contamination levels were 1.43% in PBMCV2, 0.56% in PBMCV3, 0.34% in BrainV2, 0.03% in BrainV3, 0.23% in HeartV2, and 0.12% in HeartV3 (Fig. 6d, Additional file 1: Figure S8). Therefore, the V3 datasets had lower contamination levels than V2 datasets, indicating that improvement in experimental protocols can decrease overall contamination levels. Furthermore, PBMCs exhibited over twofold higher levels of contamination compared to the brain and heart datasets suggesting that contamination levels may also depend on cell types being examined. Overall, these results demonstrate that DecontX can measure contamination from ambient RNA or other sources in the library preparation process and will be a useful method to assess the quality of different experimental protocols.

## Discussion

We developed a Bayesian method called DecontX to estimate the percentage of cross-contamination within each cell due to ambient RNA or other experimental factors. In our model, each cell is treated as a mixture of multinomial distributions over genes, one from its native cell population and another from contamination. For each cell population, the contamination distribution is defined as a combination of gene counts from other cell populations. Genes that are more highly expressed (i.e., have more UMI counts) in other cell populations will be more likely to contribute to contaminaion in the current cell population. Therefore, these genes will have relatiely higher probabilities in the contamination distribution compared to the expression distribution in native cell population and counts for these genes will be more likely to be called “contamination.” Certain types of housekeeping genes, such as ribosomal protein coding genes, can be highly expressed across many cell populations and thus will have high probabilities appearing in both the native cell populations and contamination distributions. In this case, counts for these genes will predominantly be called “native” assuming the overall proportion of estimated contamination in that cell is also relatively low. We demonstrated the accuracy of DecontX by showing it was able to accurately estimate the percentage of exogenous, contaminating transcripts in a mouse-human mixture dataset. Furthermore, after estimating and removing contaminated transcripts in 4K PBMC data, the profiles of key marker genes for each subpopulation better resembled those from sorted, purified PBMCs.

We observed that cell types with higher total mRNA abundance and prevalence within the dataset are more likely to contribute to the contamination in other cell types. For example, in the human-mouse mixture dataset, the human cells had more uniquely aligning UMIs on average than mouse cells. Therefore, the human cells contributed more reads to the ambient RNA and resulted in more contamination in mouse cells. In the 4K PBMC data, the monocytes were the second most common cell type and had the highest total numbers of UMI counts on average. In addition, monocyte-associated genes such as LYZ, S100A8, and S100A9 were the most highly expressed cell type-specific marker genes and thus contributed more to the contamination in other cell types compared to markers from other populations. These results show that contamination distributions in each cell will be dependent on the other cell types in the assay as well as the level of expression of specific genes in those cell types.

In some cases, DecontX was not able to completely remove aberrant expression of cell type markers. For example, 43.03% of NK cells in the 4K PBMC still exhibited expression of T cell markers. One potential explanation is that some cells in this dataset were actually NKT cells and truly share expression features from both NK and T cell populations [19]. Another factor is that for cell populations that share substantial similarities in gene expression patterns, DecontX will tend to be conservative and treat the majority of these counts as native expression rather than contamination. In general, we believe this behavior is desired so true biological variation between cell types is not removed. The cells estimated to be highly contaminated by DecontX in 4K PBMC were also estimated to be doublets by independent algorithms. Therefore, high contamination levels may also be useful as a quality control criterion for excluding cells. Additionally, DecontX estimation on benchmark datasets shows 10X Chromium has lowest contamination, while CEL-seq2 has highest contamination. CEL-seq2 has shown much higher frequencies of intronic and intergenic mappings compared to other scRNA-seq methods [20]. Although the SORT-seq protocol is similar to CEL-seq2, it used vapor-leak oil to prevent evaporation [7] which resulted in less estimated contaminations overall in our analysis.

By utilizing raw counts for estimation of the multinomial distributions, DecontX eliminates the potential variability that could be introduced by different normalization methods. One limitation is that cell cluster labels are needed a priori. While we automatically use Celda to identify cell clusters if none are supplied, any fast cell clustering approach can be utilized. As the contamination distribution for each cell population is derived from all other populations present in the dataset, it may sometimes better to use broader cell population labels. For example, including all T cells in one cluster rather than treating individual T cell subpopulation as a separate subcluster may help alleviate T cell-specific counts in the calculation of the contamination distributions for all T cells.

## Conclusions

DecontX can be used as an important quality assessment tool that estimates the levels of background RNA contributing to the contamination occurring as a result of dissociation procedures or other library preparation processes. Overall, computational decontamination of single-cell counts with DecontX will aid in downstream clustering and visualization and can be systematically included in analysis workflows.

## Methods

### Statistical model

We assume there are *K* known distinct cell populations among the *M* cell samples, where cell *j* has *N*_*j*_ observed transcripts. We denote native expression distribution for cell population *k* as a G-length vector *ϕ*_*k*_. For the notational convenience, we will use ***ϕ***_−*k*_={*ϕk*′:*k*^′^≠*k*,*k*^′^∈{1,2,...,*K*}} to represent gene expressions from all other cell populations other than *k*. Each cell *j* has a parameter *θ*_*j*_ to represent the proportion of transcript counts that are derived from native expression distribution. *θ*_*j*_ is assumed to come from a global beta distribution which leverages the variation of contamination level across all the cells in the dataset, with hyperparameters *a*_1_ and *a*_2_ a priori. The *t*th transcript *x*_*jt*_ in cell *j* has a hidden state, *y*_*jt*_, which follows a Bernoulli distribution parameterized by *θ*_*j*_ and denotes the transcript’s membership to native expression distribution (*y*_*jt*_=1) or contamination distribution (*y*_*jt*_=0). Assuming that transcripts are conditionally independent given hidden state *y*_*jt*_ and cell’s population *z*_*j*_,*x*_*jt*_ follows a multinomial distribution either parameterized by $\phi _{z_{j} }$ denoting native expression or $\boldsymbol {\phi }_{-z_{j}}$ denoting contamination. The joint posterior distribution can be expressed as:

1 $$\begin{array}{@{}rcl@{}} {\begin{aligned} P(\boldsymbol{X}, \boldsymbol{Z}, \boldsymbol{Y}, \boldsymbol{\theta} | \boldsymbol{\phi}, a_{1},a_{2}) &= \prod_{j=1}^{M} p(\theta_{j} | a_{1}, a_{2}) \prod_{t=1}^{N_{j}}\\& \left(\left[ p(y_{jt}=1|\theta_{j}) \cdot p(x_{jt}=g|\phi_{z_{j} }) \right]^{I(y_{jt}=1)}\right.\\ & \left.\left[ p(y_{jt}=0|\theta_{j}) \cdot p(x_{jt}=g|\boldsymbol{\phi}_{-z_{j}}) \right]^{I (y_{jt}=0)} \right). \end{aligned}} \end{array} $$

To simplify computation work and notation, we assume the contamination distribution *η*_*k*_ is a simple linear combination of ***ϕ***_−*k*_, such that:

2 $$\begin{array}{@{}rcl@{}} \eta_{k} &=& \sum_{k': k' \neq k} w_{k'} \phi_{k'}, \end{array} $$

where the weight $\phantom {\dot {i}\!}w_{k^{\prime }}$ is the proportion of native transcripts from cluster *k*^′^ and is calculated using expected values, of which the full definition is given later in inference. DecontX model construction is shown in the plate diagram:


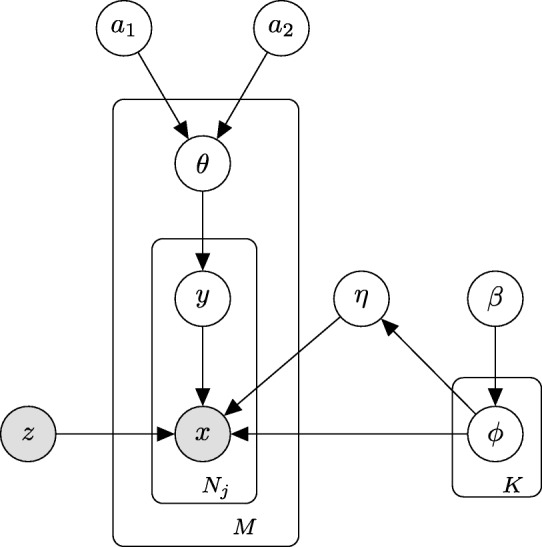


#### Variational inference

We use variational inference [21] to approximate the posterior probability of our model.

Similar to LDA [14], the following variational distributions are introduced to break down the coupling of ***θ*** and ***Y*** for variational inference:

3 $$\begin{array}{@{}rcl@{}} \begin{aligned} q (\boldsymbol{\theta}, \boldsymbol{Y} | \boldsymbol{\gamma}, \boldsymbol{\pi}) &= \prod_{j=1}^{M} q(\theta_{j} | \gamma_{j}) \prod_{t=1}^{N_{j}} q(y_{jt} | \pi_{jt}), \end{aligned} \end{array} $$

where the beta parameter *γ*_*j*_={*γ*_*j*1_,*γ*_*j*2_} and Bernoulli parameter *π*_*jt*_={*π*_*jt*1_,*π*_*jt*2_} are the free variational parameters. *π*_*jt*_ satisfies *π*_*jt*1_+*π*_*jt*2_=1, and $ q(y_{jt}) = \pi _{jt1}^{ I(y_{jt} =1)} \pi _{jt2}^{ I(y_{jt} =0)} $. The variational beta distribution for *θ*_*j*_ is $q(\theta _{j}) = \frac {\Gamma (\gamma _{j1} + \gamma _{j2}) }{\Gamma (\gamma _{j1}) \Gamma (\gamma _{j2}) } \theta _{j1}^{\gamma _{j1} -1} \theta _{j2}^{\gamma _{j2} -1} $.

The need to compute the expectation of the *θ*_*j*_ arises in deriving the variational inference. Using the general fact for exponential family that the derivative of the log normalization factor with respect to the natural parameter is equal to the expectation of the sufficient statistic (log*θ*_*ji*_,*i*∈{1,2} in our beta distribution), we have:

4 $$\begin{array}{@{}rcl@{}} \begin{aligned} E [ \log \theta_{ji} | \gamma_{j1}, \gamma_{j2} ] = \Psi (\gamma_{ji}) - \Psi (\gamma_{j1} + \gamma_{j2}), i \in \{1, 2\}, \end{aligned} \end{array} $$

where *Ψ* is the digamma function, the first derivative of the log gamma function.

For simplicity in notation, let us use *Q*={***θ***,***Y***} and *a*={*a*_1_,*a*_2_}. We begin variational inference by bounding the log-likelihood using Jensen’s inequality.

5 $$\begin{array}{@{}rcl@{}} {\begin{aligned} \log p(\boldsymbol{X}, \boldsymbol{Z} | {a}, \boldsymbol{ \phi}) &= \log \int_{Q} p(\boldsymbol{X}, \boldsymbol{Z}, \boldsymbol{\theta}, \boldsymbol{Y} | a, \boldsymbol{ \phi}) d Q\\ & = \log \int_{Q} \frac{ p(\boldsymbol{X}, \boldsymbol{Z}, \boldsymbol{\theta}, \boldsymbol{Y} | a, \boldsymbol{\phi}) } { q (\boldsymbol{\theta}, \boldsymbol{Y} | \boldsymbol{\gamma}, \boldsymbol{\pi})} q(\boldsymbol{\theta}, \boldsymbol{Y} | \boldsymbol{\gamma}, \boldsymbol{\pi})) d Q \\ & \ge \int_{Q} \log \frac{ p(\boldsymbol{X}, \boldsymbol{Z}, \boldsymbol{\theta}, \boldsymbol{Y} | a, \boldsymbol{\phi}) } { q (\boldsymbol{\theta}, \boldsymbol{Y} | \boldsymbol{\gamma}, \boldsymbol{\pi})} q(\boldsymbol{\theta}, \boldsymbol{Y} | \boldsymbol{\gamma}, \boldsymbol{\pi})) d Q \\ & = E_{Q} [\log p(\boldsymbol{X}, \boldsymbol{Z}, \boldsymbol{\theta}, \boldsymbol{Y} |a, \boldsymbol{\phi}) ] - E_{Q} [\log q (\boldsymbol{\theta}, \boldsymbol{Y} | \boldsymbol{\gamma}, \boldsymbol{\pi}) ]. \end{aligned}} \end{array} $$

Jensen’s inequality provides us with a lower bound on the log likelihood for an arbitrary variational distribution *q*(***θ***,***Y***|***γ***,***π***).

We then expand the lower bound:

6 $$\begin{array}{@{}rcl@{}} \begin{aligned} L(\boldsymbol{\gamma}, \boldsymbol{\pi}; a, \boldsymbol{ \phi}) &= E_{Q} \left[ \log p(\boldsymbol{X}, \boldsymbol{Z}, \boldsymbol{\theta}, \boldsymbol{Y} |a, \boldsymbol{\phi}) \right] \\&\quad- E_{Q} \left[\log q (\boldsymbol{\theta}, \boldsymbol{Y} | \boldsymbol{\gamma}, \boldsymbol{\pi}) \right] \\ &= E_{Q} \left[ \log p(\boldsymbol{\theta} | a) + \log p(\boldsymbol{Y} | \boldsymbol{\theta})\right. \\ &\quad \left.+ \log p(\boldsymbol{X}, \boldsymbol{Z} | \boldsymbol{Y}, \boldsymbol{\phi}) \right] \\ & \quad - E_{Q} \left[ \log q(\boldsymbol{\theta} | {\boldsymbol{\gamma}}) + \log q(\boldsymbol{Y} | \boldsymbol{\pi}) \right]. \end{aligned} \end{array} $$

Expanding each term in the lower bound by taking expectation with respect to *q*(***θ***,***Y***|***γ***,***π***):

7 $$\begin{array}{@{}rcl@{}} {\begin{aligned} E_{Q} \left[ \log p(\boldsymbol{\theta} | a) \right] &= E_{Q} \left[ \log \prod_{j=1}^{M} p (\theta_{j} | a) \right] \\&= E_{Q} \left[ \sum_{j=1}^{M} \log p(\theta_{j} |a) \right] = \sum_{j=1}^{M} E_{Q} \left[ \log p(\theta_{j} |a) \right] \\ &= \sum_{j=1}^{M} E_{Q} \left[ \log \Gamma(a_{1}+a_{2}) - \log \Gamma(a_{1}) \right.\\& \left.- \log \Gamma(a_{2}) +(a_{1}-1) \log \theta_{j1} + (a_{2}-1) \log \theta_{j2} \right] \\ &= \sum_{j=1}^{M} \left[ \log \Gamma(a_{1}+a_{2}) -\left(\sum_{i=1}^{2} \log \Gamma(a_{i}) \right)\right. \\ & \left.+\sum_{i=1}^{2}(a_{i}-1) \left(\Psi \left(\gamma_{ji} \right) - \Psi \left(\gamma_{j1}+\gamma_{j2} \right) \right) \right] \end{aligned}} \end{array} $$

8 $$\begin{array}{@{}rcl@{}} {\begin{aligned} E_{Q} \left[ \log p(\boldsymbol{Y} | \boldsymbol{\theta}) \right] &= E_{Q} \left[ \log \prod_{j=1}^{M} \prod_{t=1}^{N_{j}} p(y_{jt} | \theta_{j}) \right] \\&= E_{Q} \left[ \sum_{j=1}^{M} \sum_{t=1}^{N_{j}} \log p(y_{jt} | \theta_{j}) \right] \\ & = \sum_{j=1}^{M} \sum_{t=1}^{N_{j}} E_{Q} \left[ \log p(y_{jt} | \theta_{j}) \right] \\ &= \sum_{j=1}^{M} \sum_{t=1}^{N_{j}} E_{Q} \left[ y_{jt} \log \theta_{j1} + (1-y_{jt}) \log \theta_{j2} \right] \\ &= \sum_{j=1}^{M} \sum_{t=1}^{N_{j}} \left[ \pi_{jt1} \left(\Psi(\gamma_{j1}) - \Psi(\gamma_{j1}+\gamma_{j2}) \right) \right.\\& \left.+ \pi_{jt2} \left(\Psi(\gamma_{j2}) - \Psi(\gamma_{j1}+\gamma_{j2}) \right) \right] \end{aligned}} \end{array} $$

9 $$\begin{array}{@{}rcl@{}} {\begin{aligned} E_{Q} \left[ \log p(\boldsymbol{X}, \boldsymbol{Z} | \boldsymbol{Y}, \boldsymbol{\phi}) \right] &= E_{Q} \left[ \log \prod_{j=1}^{M} \prod_{t=1}^{N_{j}} p(x_{jt}, z_{j} | y_{jt}, \phi_{z_{j}},\eta_{z_{j}}) \right] \\ & = \sum_{j=1}^{M} \sum_{t=1}^{N_{j}} E_{Q} \left[ \log p(x_{jt}, z_{j} | y_{jt}, \phi_{z_{j}},\eta_{z_{j}}) \right]\\ &= \sum_{j=1}^{M} \sum_{t=1}^{N_{j}} E_{Q} \left[ \sum_{g=1}^{G} x_{jt}^{g} y_{jt} \log \phi_{z_{j},g} \right.\\ &\quad \left.+ x_{jt}^{g} (1- y_{jt}) \log \eta_{z_{j},g} \right] \\&= \sum_{j=1}^{M} \sum_{t=1}^{N_{j}} \sum_{g=1}^{G} E_{Q} \left[ x_{jt}^{g} y_{jt} \log \phi_{z_{j},g} \right.\\& \quad \left.+ x_{jt}^{g} (1- y_{jt}) \log \eta_{z_{j},g} \right] \\ &= \sum_{j=1}^{M} \sum_{t=1}^{N_{j}} \sum_{g=1}^{G} \left[ x_{jt}^{g} \pi_{jt1} \log \phi_{z_{j},g} \right. \\&\quad \left.+ x_{jt}^{g} \pi_{jt2} \log \eta_{z_{j},g} \right] \end{aligned}} \end{array} $$

10 $$\begin{array}{@{}rcl@{}} {\begin{aligned} E_{Q} \left[ \log q(\boldsymbol{ \theta} | \boldsymbol{ \gamma}) \right] &= E_{Q} \left[ \log \prod_{j=1}^{M} q(\theta_{j} | \gamma_{j}) \right] \\&= E_{Q} \left[ \sum_{j=1}^{M} \log q(\theta_{j} | \gamma_{j}) \right] = \sum_{j=1}^{M} E_{Q} \left[ \log q(\theta_{j} | \gamma_{j}) \right] \\ &= \sum_{j=1}^{M} E_{Q} \left[ \log \Gamma \left(\gamma_{j1} + \gamma_{j2} \right) - \log \Gamma (\gamma_{j1}) \right.\\ &- \log \Gamma (\gamma_{j2}) + \left(\gamma_{j1} -1 \right) \log \theta_{j1} \\ & \left. + \left(\gamma_{j2} -1 \right) \log \theta_{j2} \right] \\ &= \sum_{j=1}^{M} \left[ \log \Gamma \left(\gamma_{j1} + \gamma_{j2} \right) - \left(\sum_{i=1}^{2}\log \Gamma (\gamma_{ji}) \right) \right. \\& \left.+\sum_{i=1}^{2} \left(\gamma_{ji} -1 \right) (\Psi (\gamma_{ji}) - \Psi (\gamma_{j1} + \gamma_{j2})) \right] \end{aligned}} \end{array} $$

11 $$\begin{array}{@{}rcl@{}} {\begin{aligned} E_{Q} \left[ \log q(\boldsymbol{ Y} | \boldsymbol{ \pi}) \right] &= E_{Q} \left[ \log \prod_{j=1}^{M} \prod_{t=1}^{N_{j}} q(y_{jt} | \pi_{jt}) \right] \\&\quad= E_{Q} \left[ \sum_{j=1}^{M} \sum_{t=1}^{N_{j}} \log q(y_{jt} | \pi_{jt}) \right] \\ & = \sum_{j=1}^{M} \sum_{t=1}^{N_{j}} E_{Q} \left[ \log q(y_{jt} | \pi_{jt}) \right] \\ &= \sum_{j=1}^{M} \sum_{t=1}^{N_{j}} E_{Q} \left[ y_{jt} \log \pi_{jt1} + (1 - y_{jt}) \log \pi_{jt2} \right] \\ &= \sum_{j=1}^{M} \sum_{t=1}^{N_{j}} \left[ \pi_{jt1} \log \pi_{jt1} + \pi_{jt2} \log \pi_{jt2} \right]. \end{aligned}} \end{array} $$

We then maximize the lower bound with respect to the variational parameters ***γ*** and ***π***.

First we maximize the lower bound with respect to ***π***. Since (*π*_*jt*_)s are independent, for *t*∈{1,2,...,*N*_*j*_}, we isolate the terms that contains *π*_*jt*_. Lagrangian multiplier is added due to the constraint *π*_*jt*1_+*π*_*jt*2_=1. We substituted $x_{jt}^{g} \pi _{jt1} \log \phi _{z_{j},g}$ and $x_{jt}^{g} \pi _{jt2} \log \eta _{z_{j},g}$ from Eq. 9 with $ \pi _{jt1} \log \phi _{z_{j},g}$ and $\pi _{jt2} \log \eta _{z_{j},g}$, respectively, since $ x_{jt}^{g} = I(x_{jt} =g) $ and is observed:

12 $$\begin{array}{@{}rcl@{}} \begin{aligned} L_{[ \pi_{jt} ]} &= \left[ \pi_{jt1} \left(\Psi(\gamma_{j1}) - \Psi(\gamma_{j1}+\gamma_{j2}) \right)\right. \\&\quad \left.+ \pi_{jt2} \left(\Psi(\gamma_{j2}) - \Psi(\gamma_{j1}+\gamma_{j2}) \right) \right] \\ & \quad + \left[ \pi_{jt1} \log \phi_{z_{j},g} + \pi_{jt2} \log \eta_{z_{j},g} \right] \\ & \quad - \left[ \pi_{jt1} \log \pi_{jt1} + \pi_{jt2} \log \pi_{jt2} \right] \\ & \quad - \lambda (\pi_{jt1} + \pi_{jt2} - 1). \end{aligned} \end{array} $$

Taking derivative with respect to *π*_*jt*1_, we obtain:

13 $$\begin{array}{@{}rcl@{}} \begin{aligned} \frac{\partial L }{\partial \pi_{jt1}} & = \left(\Psi(\gamma_{j1}) - \Psi(\gamma_{j1}+\gamma_{j2}) \right) \\&\quad+ \log \phi_{z_{j},g} -\log \pi_{jt1} - \lambda - 1. \end{aligned} \end{array} $$

Setting this derivative to zero yields the maximizing value of the variational parameter *π*_*jt*1_:

14 $$\begin{array}{@{}rcl@{}} \begin{aligned} \pi_{jt1} \propto \phi_{z_{j},g} \exp (\Psi(\gamma_{j1}) - \Psi(\gamma_{j1}+\gamma_{j2})). \end{aligned} \end{array} $$

Similarly, we could have *π*_*jt*2_:

15 $$\begin{array}{@{}rcl@{}} \begin{aligned} \pi_{jt2} \propto \eta_{z_{j},g} \exp (\Psi(\gamma_{j2}) - \Psi(\gamma_{j1}+\gamma_{j2})). \end{aligned} \end{array} $$

Next, we maximize the lower bound with respect to ***γ***. Since (*γ*_*j*_)s are independent for *j*∈1,2,...,*M*, each *γ*_*j*_ can be estimated separately. We isolate the terms that contain *γ*_*j*_.

16 $$\begin{array}{@{}rcl@{}} \begin{aligned} L_{\left[ \gamma_{j} \right]} &= \sum_{i=1}^{2}(a_{i}-1) \left(\Psi \left(\gamma_{ji} \right) - \Psi \left(\gamma_{j1}+\gamma_{j2} \right) \right) \\ & + \sum_{t=1}^{N_{j}} \left[ \pi_{jt1} \left(\Psi(\gamma_{j1}) - \Psi(\gamma_{j1}+\gamma_{j2}) \right)\right. \\& \left. \quad + \pi_{jt2} \left(\Psi(\gamma_{j2}) - \Psi(\gamma_{j1}+\gamma_{j2}) \right) \right] \\ & - \left[ \log \Gamma \left(\gamma_{j1} + \gamma_{j2} \right) - \left(\sum_{i=1}^{2}\log \Gamma (\gamma_{ji}) \right) \right.\\& \left. +\sum_{i=1}^{2} \left(\gamma_{ji} -1 \right) (\Psi (\gamma_{ji}) - \Psi (\gamma_{j1} + \gamma_{j2})) \right]. \end{aligned} \end{array} $$

Taking derivative with respect to *γ*_*ji*_, we obtain:

17 $$\begin{array}{@{}rcl@{}} \begin{aligned} \frac{ \partial L }{ \partial \gamma_{ji} } & = \Psi ' (\gamma_{ji}) \left(a_{i} + \sum_{t=1}^{N_{j}} \pi_{jt1} -\gamma_{j1} \right) - \Psi ' (\gamma_{j1} + \gamma_{j2})\\&\quad \left(a_{1} + \sum_{t=1}^{N_{j}} \pi_{jt1} -\gamma_{j1} + a_{2} + \sum_{t=1}^{N_{j}} \pi_{jt2} -\gamma_{j2} \right), \end{aligned} \end{array} $$

where *Ψ*^′^ is the derivative of the digamma function. Setting this derivative to zero yields a maximum at:

18 $$\begin{array}{@{}rcl@{}} \begin{aligned} \gamma_{ji} = a_{i} + \sum_{t=1}^{N_{j}} \pi_{jt1}, i \in \{ 1,2 \}. \end{aligned} \end{array} $$

Finally, we move forward to estimating ***ϕ*** and *a*, and to update ***η***.

To maximize with respect to *ϕ*_*k*_, we isolate terms and add Lagrangian multiplier due to the constraint $\sum _{g=1}^{G} \phi _{kg} = 1$:

19 $$\begin{array}{@{}rcl@{}} \begin{aligned} L_{[\phi_{k}]} &= \sum_{j: z_{j} = k} \sum_{t=1}^{N_{j}} \sum_{g=1}^{G} x_{jt}^{g} \pi_{jt1} \log \phi_{kg} - \lambda (\sum_{g=1}^{G} \phi_{kg} -1). \end{aligned} \end{array} $$

Taking the derivative with respect to *ϕ*_*kg*_ and set it to zero, we get:

20 $$\begin{array}{@{}rcl@{}} \begin{aligned} \phi_{kg} \propto \sum_{j: z_{j}=k} \sum_{t=1}^{N_{j}} x_{jt}^{g} \pi_{jt1}. \end{aligned} \end{array} $$

The weight $\phantom {\dot {i}\!}w_{k^{\prime }}$ is the proportion of native transcripts from cluster *k*^′^ and is calculated using expected values:

21 $$\begin{array}{@{}rcl@{}} \begin{aligned} w_{k'} = \frac{ \sum_{k': k' \neq k} \left(\sum_{j: z_{j} = k'} \sum_{t=1}^{N_{j}} \pi_{jt1} \right) }{ \sum_{j: z_{j} \neq k} \sum_{t=1}^{N_{j}} \pi_{jt1} }. \end{aligned} \end{array} $$

Hence, we have our updated $\phantom {\dot {i}\!}\eta _{k^{\prime }g}$ as:

22 $$\begin{array}{@{}rcl@{}} \begin{aligned} \eta_{kg} &= \frac{\sum_{k': k' \neq k} \left(\sum_{j: z_{j} = k'} \sum_{t=1}^{N_{j}} \pi_{jt1} \right) \phi_{k'g}} { \sum_{j: z_{j} \neq k} \sum_{t=1}^{N_{j}} \pi_{jt1} } \\ &= \frac{ 1} { \sum_{j: z_{j} \neq k} \sum_{t=1}^{N_{j}} \pi_{jt1} } \sum_{k': k' \neq k} \left(\sum_{j: z_{j} = k'} \sum_{t=1}^{N_{j}} \pi_{jt1} \right) \phi_{k'g} \\ &= \frac{ 1} { \sum_{j: z_{j} \neq k} \sum_{t=1}^{N_{j}} \pi_{jt1} } \sum_{k': k' \neq k} \left(\sum_{j: z_{j} = k'} \sum_{t=1}^{N_{j}} \pi_{jt1} \right)\\&\quad \frac{\sum_{j: z_{j}=k'} \sum_{t=1}^{N_{j}} x_{jt}^{g} \pi_{jt1}}{\sum_{j: z_{j} = k'} \sum_{t=1}^{N_{j}} \pi_{jt1}} \\ &= \frac{ 1} { \sum_{j: z_{j} \neq k} \sum_{t=1}^{N_{j}} \pi_{jt1} } \sum_{k': k' \neq k} \left(\sum_{j: z_{j}=k'} \sum_{t=1}^{N_{j}} x_{jt}^{g} \pi_{jt1} \right) \\ &= \frac{ \sum_{k': k' \neq k} \left(\sum_{j: z_{j}=k'} \sum_{t=1}^{N_{j}} x_{jt}^{g} \pi_{jt1} \right) } { \sum_{j: z_{j} \neq k} \sum_{t=1}^{N_{j}} \pi_{jt1} }. \end{aligned} \end{array} $$

To maximize with respect to *a*, we isolate terms and get:

23 $$\begin{array}{@{}rcl@{}} \begin{aligned} L_{[a]} &= \sum_{j=1}^{M} \left[ \log \Gamma(a_{1}+a_{2}) - \left(\sum_{i=1}^{2}\log \Gamma(a_{1}) \right)\right. \\ &\quad \left. +\sum_{i=1}^{2}(a_{i}-1) \left(\Psi \left(\gamma_{ji} \right) - \Psi \left(\gamma_{j1}+\gamma_{j2} \right) \right) \right]. \end{aligned} \end{array} $$

A Newton iteration can be used to find the maximal point *a* [22], which requires both the first and second derivatives of *L*_[*a*]_. The first derivative, gradient ∇*L*, and the second derivative, Hessian matrix *H,* are:

24 $$\begin{array}{@{}rcl@{}} \begin{aligned} \nabla L_i = \frac{ \partial L_{[a]} }{ \partial a_{i}} &= \sum_{j=1}^{M} \left(\Psi (a_{1} + a_{2}) - \Psi (a_{i}) \right.\\ &\quad \left.+ \Psi (\gamma_{ji}) - \Psi \left(\gamma_{j1}+\gamma_{j2} \right) \right) \end{aligned} \end{array} $$

25 $$\begin{array}{@{}rcl@{}} \begin{aligned} & H_{ii} = \frac{ \partial^{2} L_{[a]} }{ \partial a_{i}^{2} } = M \left(\Psi '(a_{1} + a_{2}) - \Psi (a_{i}) \right), i \in \{1,2\} \\ & H_{ij} = \frac{ \partial^{2} L_{[a]} }{ \partial a_{i} \partial a_{j} } = M \Psi '(a_{1} + a_{2}), j \neq i. \end{aligned} \end{array} $$

One Newton step is then:

26 $$\begin{array}{@{}rcl@{}} \begin{aligned} a^{new} = a^{old} - H^{-1} \nabla L. \end{aligned} \end{array} $$

### Analysis of sorted human-mouse mixture single-cell dataset

A mixture of fresh frozen human (HEK293T) and mouse (NIH3T3) cells were sequenced together in 10X Genomics Chromium. This data is available at 10X Genomics website [23]. A total of 6164 human cells, 5915 mouse cells, and 741 multiplets were detected by CellRanger. Excluding multiplets, 12,079 cells with CellRanger-predicted cell type were used to estimate contamination using DecontX.

### Analysis of sorted PBMCs single-cell datasets

Nine publicly available PBMC datasets totalling of 84,432 cells [24] were obtained from 10X Genomics. Each dataset consisted of a population of cells that were isolated with flow cytometry based on expression of a predefined protein marker. Cell populations included progenitor cells(CD34+), monocytes (CD14+), B cells (CD19+), natural killer cells (CD56+), helper T cells (CD4+), regulatory T cells (CD4+/CD25+), native T cells (CD4+/CD45RA+/CD25 −), naive cytotoxic T cells (CD8+/CD45RA+), and cytotoxic T cells (CD8+). A total of 7363 genes which contained at least 3 counts across 3 cells were included in the analysis. DecontX used cell label by flow cytometry to estimate contamination. Celda [17] was used to identify 76 gene modules and 21 cell clusters, including 8 clusters predominantly expressing T cell markers, 2 clusters predominantly expressing natural killer cell markers, 2 clusters predominantly expressing B cell markers, 2 clusters predominantly expressing monocyte markers, and 7 clusters predominantly expressing CD34 progenitor cell markers. These computationally inferred cell type labels were used in downstream analyses that examined the percentage of cells that express various marker genes. Using computationally derived cell clustered mitigated instances where a cell was improperly sorted and labeled by flow cytometry as belonging to one population when in fact it was transcriptionally similar to another population.

### Analysis of the 4K PBMC single-cell dataset

A total of 4340 PBMCs [25] from a healthy donor were sequenced in a single channel of the 10X Genomics Chromium. A total of 4529 genes which contained at least 3 counts across 3 cells were included in the analysis. Nineteen cell clusters and 150 gene modules were identified with Celda [17]. Cell clusters 2 and 3 were classified as B cells (MS4A1+); cell clusters 5, 6, 7, 8, 9, and 11 were classified as T cells (CD3D+/CD3E+); cell clusters 13 and 14 were identified as LYZ+ monocyte group; cell cluster 15 was identified as FCGR3A+ monocyte group; cell cluster 10 was identified as NKG7+ and GNLY+ NK cell group; cell clusters 18 and 19 were identified as FCER1A+ dendritic cell group; cell cluster 4 was identified as IRF7+ and IRF8+ plasmacytoid dendritic cell group; cell cluster 16 was identified as PPBP+ megakaryocytes; cell cluster 1 was identified as IGHG1+ and IGHG2+ plasma cell group; cell cluster 12 was identified as CD34+ cell group; cell cluster 17 is likely to be multiplets for it has shown IL7R, CD3D, and CD14 markers. DecontX used Celda-estimated cluster label to estimate contamination.

### Analysis of benchmark datasets

Data were generated as previously described [7] and is available at their github repository [26]. Briefly, five human lung adenocarcinoma cell lines (HCC827, H1975, A549, H838, and H2228) were cultured separately and the same batch was processed in two different ways to create three datasets. For the two single-cell datasets, single cells from three or five cell lines were mixed together, with libraries generated using three different protocols (10X Chromium, Drop-seq, CEL-seq2). For the mixRNA datasets, RNA was extracted in bulk from three cell lines (HCC827, H1975, and H2228), mixed in seven different proportions, and diluted to single-cell equivalent amounts, with libraries generated using either CEL-seq2 or SORT-seq protocols. Available cell clusters from the paper estimated by Demuxlet were used to estimate contamination by DecontX on both single-cell datasets; all cells were included for DecontX analysis. For the mixRNA datasets, we assign the cell cluster of each pseudo-cell being the predominant cell line that has contributed more than 50% of the total mRNA.

### Analysis of three tissues across two 10X Chromium platforms

Three tissue types (mouse brain, mouse heart, PBMC from healthy donor) were profiled using two different 10X 3’ protocols (V2, V3). The six datasets are available at 10X Genomics [27–32]. A total of 1206 cells were detected in BrainV2, 1301 in BrainV3, 712 in HeartV2, 1011 in HeartV3, 996 in PBMCV2, and 1222 in PBMCV3. Genes which contained at least 3 counts across 3 cells were included in the analysis. Automatic clustering is performed on each dataset. Specifically, for each dataset, genes were collapsed into 100 gene modules using Celda [17], UMAP [33] was used on the 100 gene modules to define spacial similarity between cells on a reduced two-dimensional space, and then, density-based spatial clustering of applications with noise [34] (DBSCAN) was used with parameter epsilon as 1 to define cell clusters.

## Supplementary information

**Additional file 1** Supplementary figures, including Figure S1–S8.

**Additional file 2** Review history.

## Data Availability

The human-mouse cell mixture data that support the findings of this study are available from 10X Genomics [23]. The sorted PBMC data that support the findings of this study are included in this published article *Massively parallel digital transcriptional profiling of single cells* [5]. The data are available under accession number SRP073767 in the Short Read Archive, and are also available at 10X Genomics [24]. The PBMC 4K data that support the findings of this study are available from 10X Genomics [25]. The benchmark data that support the findings of this study are included in this published article *Benchmarking Single Cell RNA-sequencing analysis pipelines using mixture control experiments* [7] and its supplementary information files, and also available at their github repository [26]. The six datasets (BrainV2, BrainV3, HeartV2, HeartV3, PBMCV2, and PBMCV3) that support the findings of this study are available at 10X Genomics [27–32]. DecontX is freely available at https://github.com/campbio/celda under MIT license. The source code used in the manuscript is deposited at Zenodo and github [35].
