# Supplementary material for: Decontamination of ambient RNA in single-cell RNA-seq with DecontX
Source: Genome Biol. 2020 Mar 5;21:57. doi: 10.1186/s13059-020-1950-6 (PMC7059395; doi:10.1186/s13059-020-1950-6)
Supplement: Supplementary file 1 — Additional file 1 Supplementary figures, including Figure S1–S8. [file 13059_2020_1950_MOESM1_ESM.pdf]

Supplementary: Decontamination of ambient RNA in single-cell RNA-seq with DecontX

Fig S1

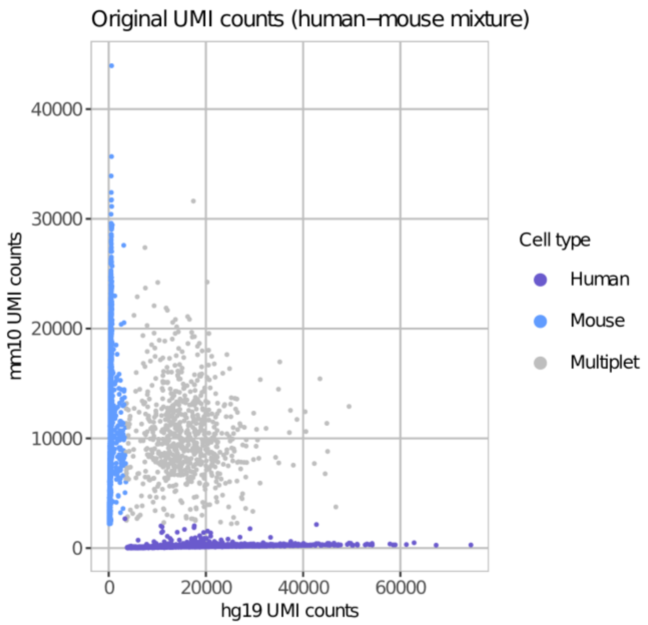

Supplementary Figure 1: Identification human and mouse cells in droplets. The number of UMIs aligned specifically to mouse genome is plotted against to the number of UMIs aligned specifically to human genome for each droplet. CellRanger was used to predict whether each droplet contained a human cell (purple), mouse cell (blue), or multiplet (grey). The multiplets were excluded from down-stream decontamination analysis.

Fig S2

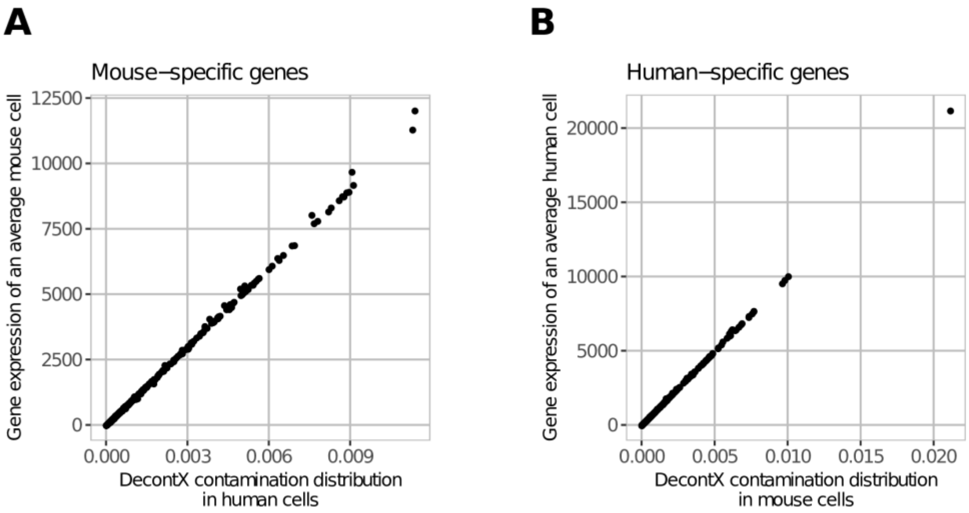

Supplementary Figure 2: Comparisons between distributions of population-specific contamination and native expression in the mouse-human mixture dataset. (A) A high correlation was observed between the gene probabilities in the DecontX-estimated contamination distribution for the human cell population and the gene expression levels within an average mouse cell. Each point represents a gene in the mouse transcriptome. **(B)** A high correlation was observed between the gene probabilities in the DecontX-estimated contamination distribution for the mouse cell population and the gene expression levels within an average human cell. Each point represents a gene in the human transcriptome.

Fig S3

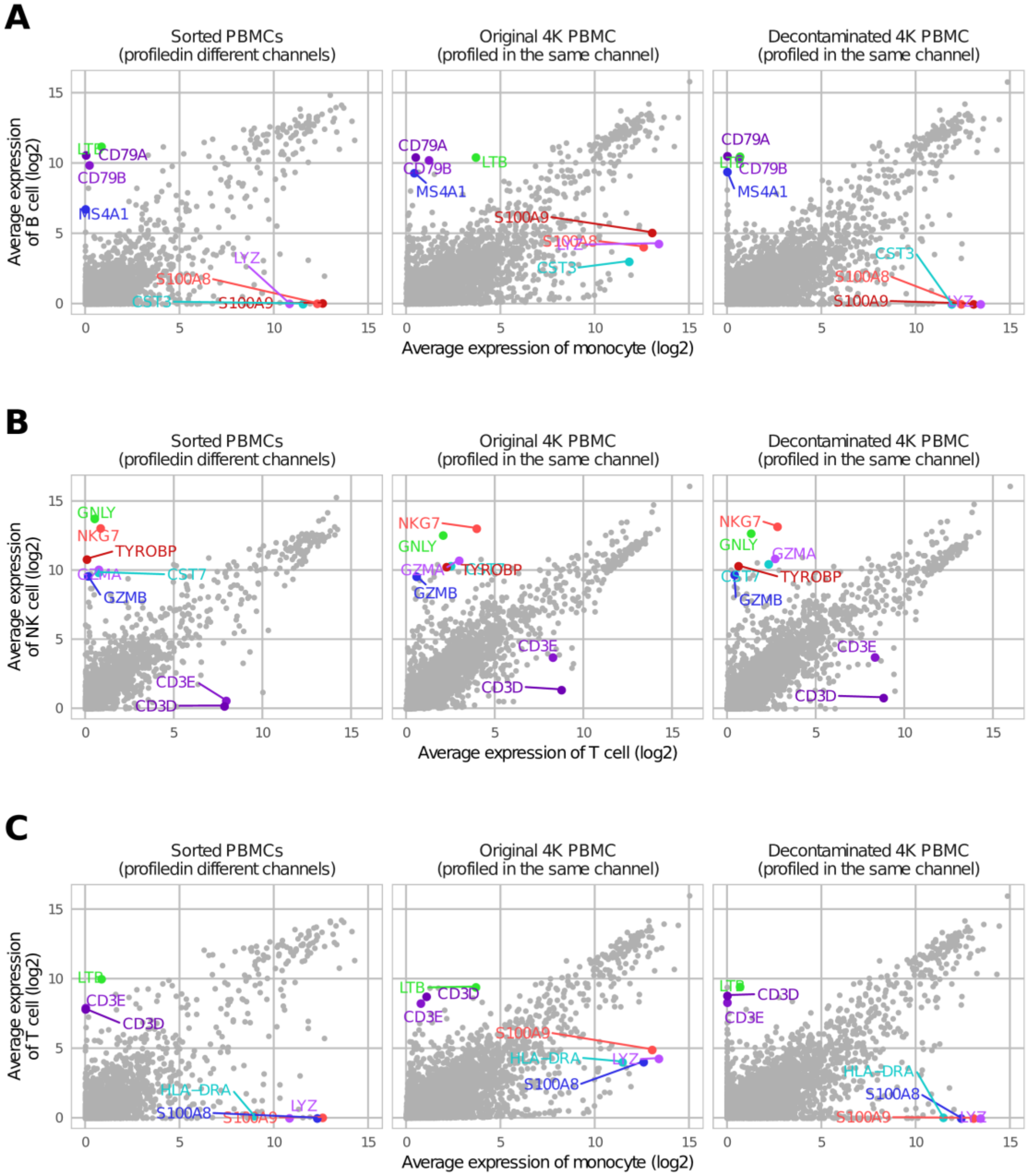
A
Sorted PBMCs
(profiledin different channels)
Original 4K PBMC
(profiled in the same channel)
Decontaminated 4K PBMC
(profiled in the same channel)
Average expression of B cell (log2)
Average expression of monocyte (log2)
B
Average expression of NK cell (log2)
Average expression of T cell (log2)
C
Average expression of T cell (log2)
Average expression of monocyte (log2)

Fig S3

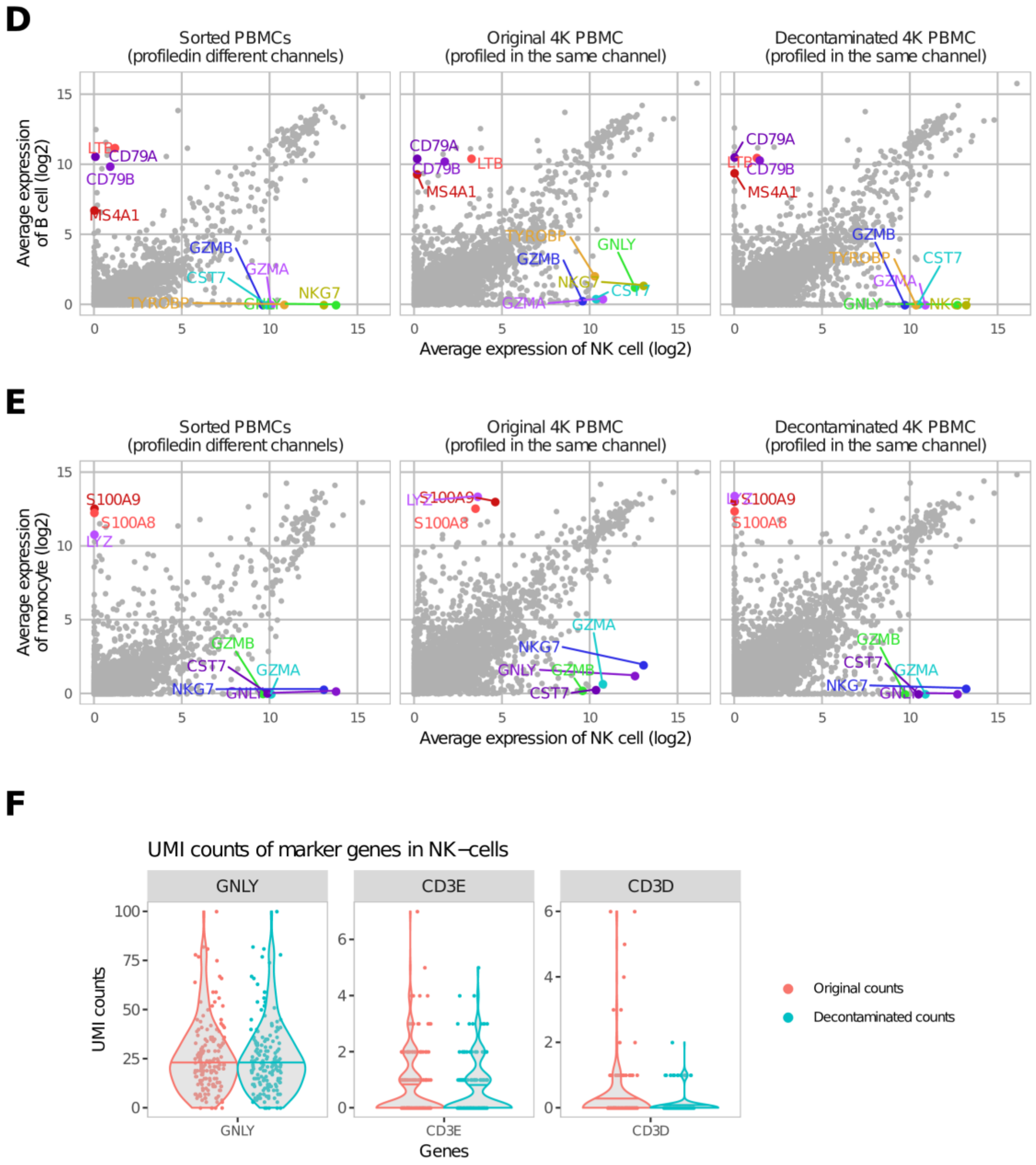

Supplementary Figure 3: Expression of PBMC cell-type specific marker genes across cell populations. For each gene, the average expression across all cells in a population is plotted against the average expression across all cells in another population. **Left:** data from sorted PMBCs profiled in different channels. **Middle:** data from the PBMC 4K before decontamination with DecontX; **Right:** PBMC 4K data after decontamination with DecontX. Comparisons of cell populations include: **(A)** B-cells vs monocytes, **(B)** NK-cells vs T-cells, **(C)** T-cells vs monocytes, **(D)** B-cells vs NK-cells, and **(E)** monocytes vs NK-cells. **(F)** Expression levels of NK-cell specific marker

gene (GNLY) and T-cell specific marker genes (CD3E and CD3D) in NK-cells before and after decontamination.

Fig S4

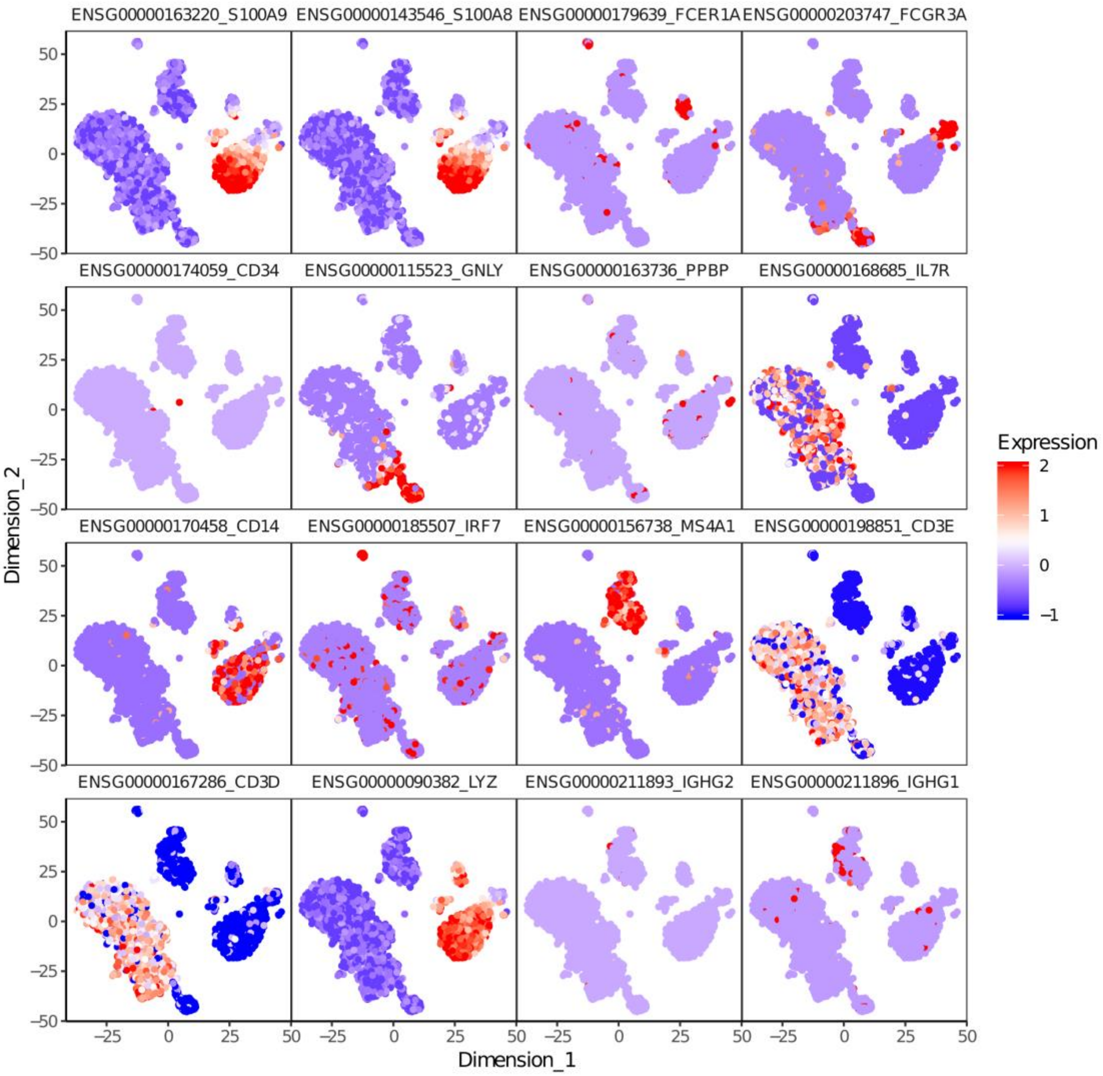

Supplementary Figure 4: Expression of PBMC markers in the 4K dataset. A tSNE was generated using the module probabilities derived from Celda. Each point is a cell and is colored by the relative expression level of the specific marker gene using the original 4k PBMC dataset before decontamination.

Fig S5

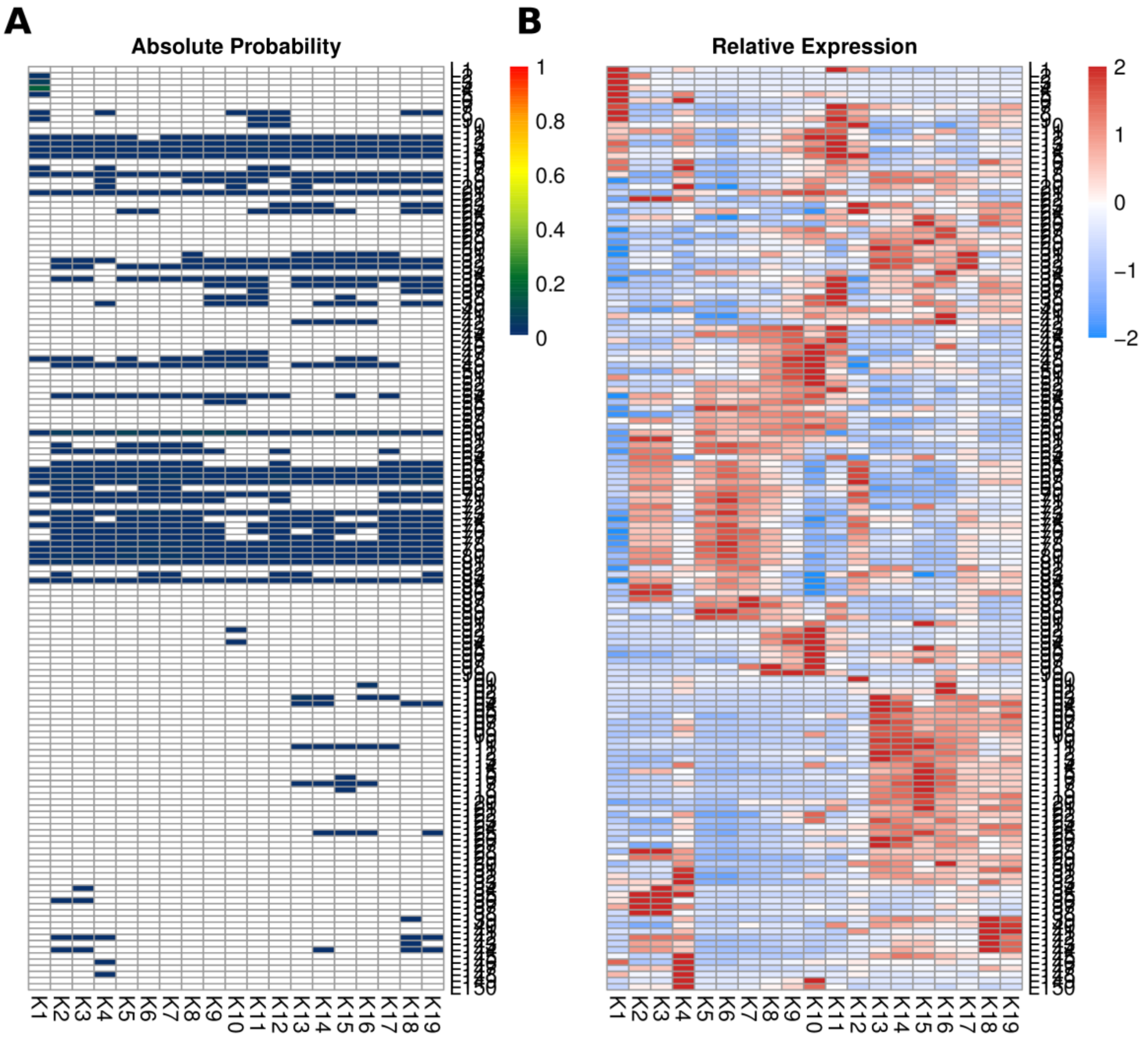

Supplementary Figure 5: Celda probability and relative expression heatmaps for PBMC 4K. (A) The probability for each of 150 modules (rows) is shown in each of the 19 cell populations (columns). **(B)** The relative expression for each of 150 module (rows) is shown for each of the 19 cell populations (columns).

Fig S6

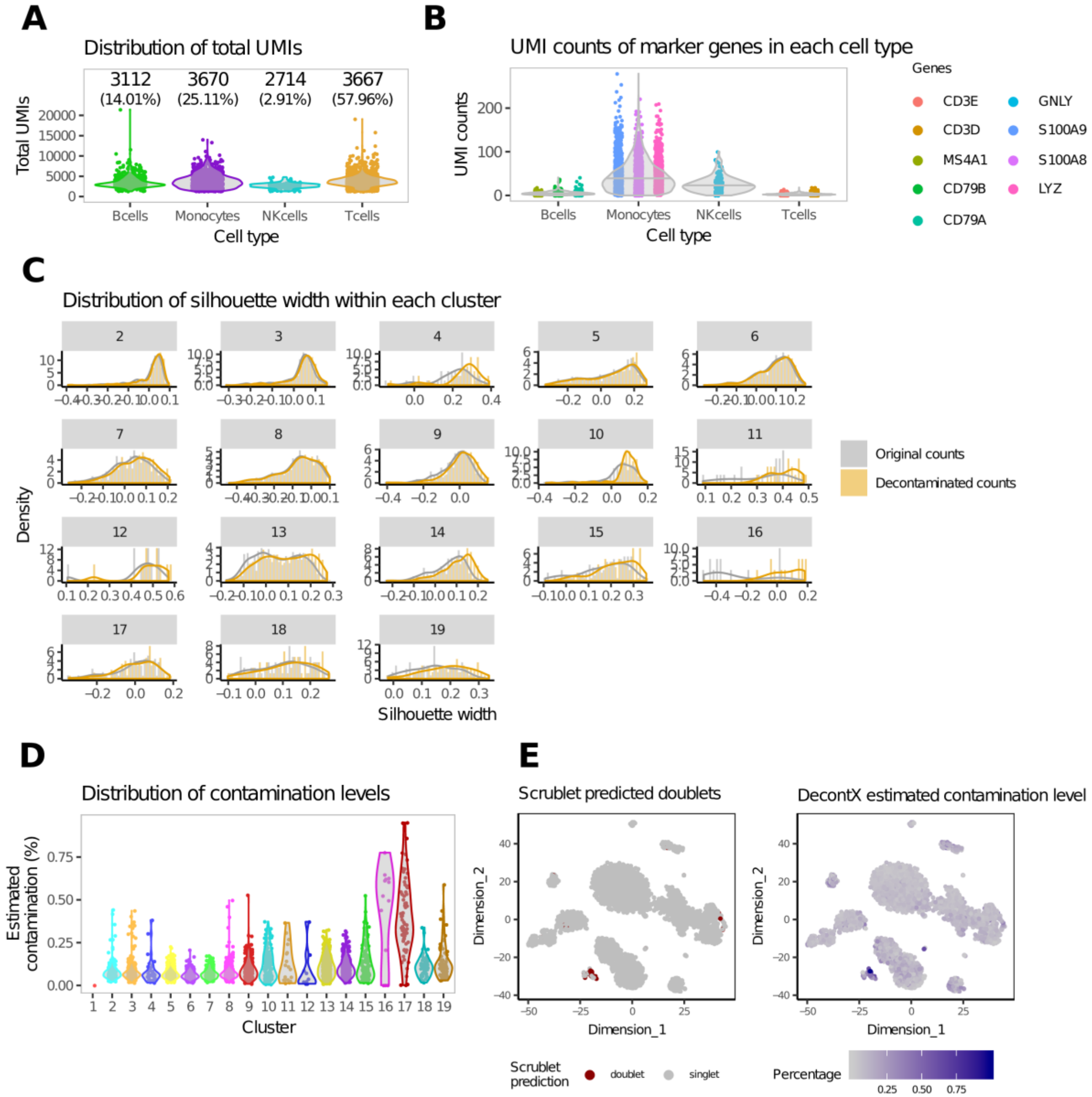

Supplementary Figure 6: Comparison of contamination levels within cell types and with predicted doublets. (A) Distribution of number of UMIs within each major cell type. Median UMIs (top) and the percentage of total UMIs in the whole dataset (bottom) are labeled for each cell type. **(B)** Distribution of number of UMIs for cell-type specific marker genes in each major cell type. **(C)** Distribution of silhouette width within each cluster on original counts (grey) and decontaminated counts (yellow). Cluster 1 has only one cell and was excluded from the plot. **(D)** Distribution of DecontX estimated contamination level within each cluster. **(E)** tSNE plots of

DecontX decontaminated PBMC 4K data. Red cells are predicted doublets by Scrublet (left), each cell is colored by contamination level estimated by DecontX (right).

Fig S7

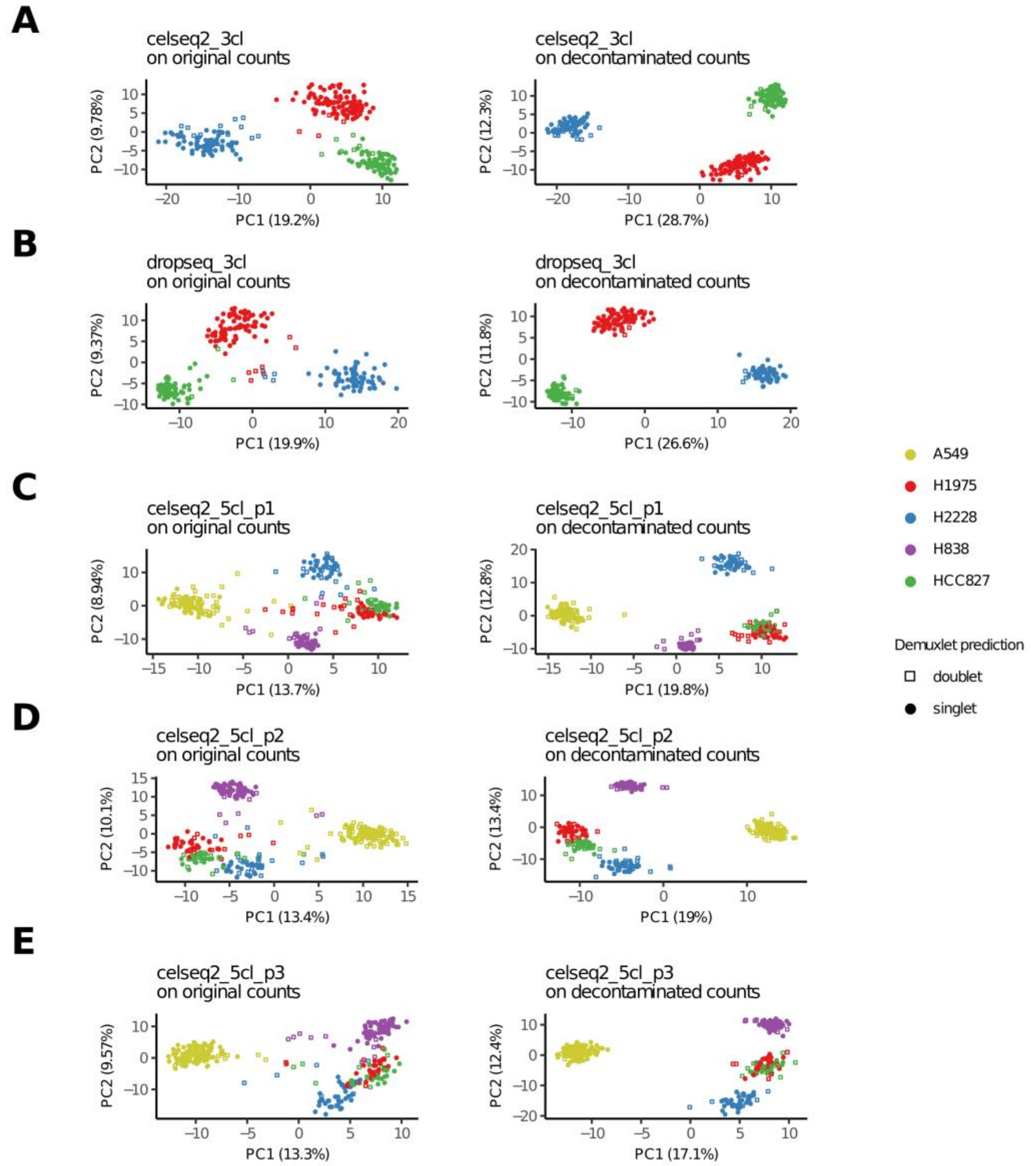

Supplementary Figure 7: Benchmark single cell datasets before and after decontamination. PCA was applied to mixed single cells from three cell lines sequenced with **(A)** CEL-seq2 or **(B)** Drop-seq**. (C, D, E)** PCA was applied to mixed single cells on both original counts (left) and decontaminated counts (right) from five cell lines sequenced in three different batches (p1, p2, p3) using CEL-seq2.

Fig S8

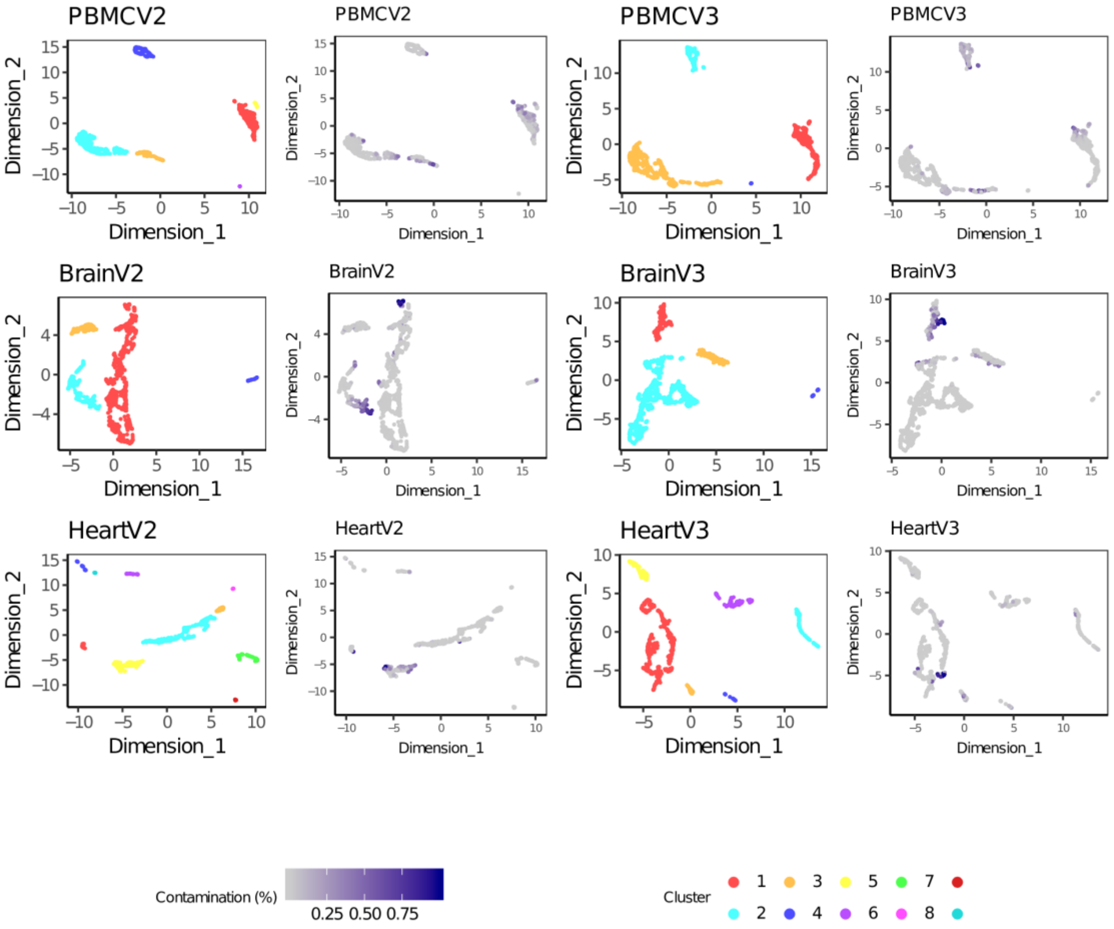

Supplementary Figure 8: Contamination levels of cell types from three different tissues profiled with two 10X protocols. UMAPs of Brain, Heart, and PBMC single cell datasets are colored by cluster label (first and third columns) or contamination levels estimated by DecontX (second and fourth columns).
