# Supplementary material for: Decontamination of ambient RNA in single-cell RNA-seq with DecontX
Source: Genome Biol. 2020 Mar 5;21:57. doi: 10.1186/s13059-020-1950-6 (PMC7059395; doi:10.1186/s13059-020-1950-6)
Supplement: Supplementary file 2 — Additional file 2 Review history. [file 13059_2020_1950_MOESM2_ESM.docx]

Review History

**First round of review**

**Reviewer 1**

**Were you able to assess all statistics in the manuscript, including the appropriateness of statistical tests used?**

Yes: I have read the statistics description and it was clearly written

**Were you able to directly test the methods?**

No

**Comments to author:**

In this manuscript, Yang et al. presented a new algorithm-DecontX to identify and remove the contaminated reads in single cell RNA-seq datasets. The authors first applied their method to a mouse-human mixture dataset and clearly demonstrated the cross-species contamination in the dataset. After that, the method was applied to a single cell RNA-seq dataset of peripheral mononuclear cells. The authors showed that the aberrant expression of marker genes can be eliminated by the method. At last, the method was applied to a published benchmark dataset to show that different single cell RNA-seq protocols have different degrees of contamination.

Overall, this paper is well organized, and the major claim can be well supported by the results presented in this paper. There are a few minor issues that need to be addressed.

1. In figure 4, the method was applied to a PBMC scRNA-seq dataset to remove the aberrant expression of marker genes. Although the aberrant expression of the marker genes of other cell types was nearly eliminated in B-cells, T-cells and monocytes, the expression of T-cell specific markers and B-cell specific markers in NK cells was not successfully removed. Hence, the performance of DecontX could be different between different cell types. If my understanding is right, I would suggest to clearly state the potential existing cell-type dependent effect.

2. In figure 5A-C, the clustering performance was evaluated before and after decontamination by DecontX, and silhouette width was calculated as a quantitative evaluation. In figure 5C, the authors only claimed that the mean silhouette width was increased from 0.04 to 0.06 after decontamination. To present the difference of silhouette width between before and after decontamination, it is better to show the histogram distribution of silhouette.

Read contamination is a widely existing problem in single cell RNA-seq field. Based on the results presented in this paper, DecontX provide the greatly desired tool for evaluating the quality of single cell RNA-seq datasets and reducing contamination levels in high-throughput RNA-seq studies. Once the authors make the changes described above, I will recommend the acceptance by GB.

**Reviewer 2**

**Were you able to assess all statistics in the manuscript, including the appropriateness of statistical tests used?**

Yes: There is no statistical problems in the manuscript.

**Were you able to directly test the methods?**

No

**Comments to author:**

The authors should introduce other algorithms of doublet removers such as scds (10.1093/bioinformatics/btz698) and Scrublet (10.1016/j.cels.2018.11.005) in the section of Introduction for the reader's understanding. The description would highlight the originality of this paper.

In this paper, the authors developed DecontX for drop-based scRNA-seq. However, in fact, there was a significant impact on CEL-seq2, which is one of the plate-based protocol. The authors more deeply and carefully should discuss the reason for the high contaminant in CEL-seq2. CEL-seq2 might use cell sorter. In general, ambient RNA cannot be contaminated in each cell when use cell sorter. The author expected the steps of library preparation is critical. Which step of library preparation do you think has the problem? Cell sorting, doublet, cell debris, contamination from aerosol, abnormal multiplexing procedure, contamination of genome sequence, or cell barcode swapping? Some papers pointed out that CEL-seq2 shows the irregular distribution of the genomic mapping region. Many of CEL-seq2's reads abnormally are map the intergenic and intron region (Fig. 1. in Elisabetta Mereu et al. bioRxiv).

In the manuscript, the contamination rate is estimated in several protocols. However, the contamination rate is affected by experimental environments such as tissue dissociation protocol and the skill of a person. The authors should investigate the variation of contamination rate among difference data by the same protocol if you claim the difference of contamination rate between each protocol.

If the graphical model of the statistical model is shown, it will help the reader understand.

In figure 2, Why is the proportion of contamination in the human cell less than that of the mouse cell?

In figure 4, why is the mixture of monocyte the majority? Could you discuss the biological or technical reasons?

In figure 5, the author should show the names of the cell type in each cluster.

Could you more deeply discuss the relationship between highly contaminated genes, gene function, expression level, and cell-type specificity of expression?

**Authors Response**

**Point-by-point responses to the reviewers’ comments:**

Reviewer #1:

 In this manuscript, Yang et al. presented a new algorithm-DecontX to identify and remove the contaminated reads in single cell RNA-seq datasets. The authors first applied their method to a mouse-human mixture dataset and clearly demonstrated the cross-species contamination in the dataset. After that, the method was applied to a single cell RNA-seq dataset of peripheral mononuclear cells. The authors showed that the aberrant expression of marker genes can be eliminated by the method. At last, the method was applied to a published benchmark dataset to show that different single cell RNA-seq protocols have different degrees of contamination.  Overall, this paper is well organized, and the major claim can be well supported by the results presented in this paper. There are a few minor issues that need to be addressed.

1. In figure 4, the method was applied to a PBMC scRNA-seq dataset to remove the aberrant expression of marker genes. Although the aberrant expression of the marker genes of other cell types was nearly eliminated in B-cells, T-cells and monocytes, the expression of T-cell specific markers and B-cell specific markers in NK cells was not successfully removed. Hence, the performance of DecontX could be different between different cell types. If my understanding is right, I would suggest to clearly state the potential existing cell-type dependent effect.

*Thank you for the suggestion as this is an important point. Even though expression of T-cell markers was not entirely removed from NK-cells, they were still substantially decreased. We make this point in the results and have added Supplementary Figure 3F. Specifically, we state (page 4, line 39):*

*“The only exception was that 43.03% of NK-cells retained some expression of T-cell markers. Despite this, overall expression of T-cell markers was till significantly reduced in NK-cells (p-value = 0.0005 for CD3D, p-value = 0.0005 for CD3E; Supplementary Figure 3F).”*

*We have also added the following statement to the discussion to explain how cell types can affect performance of DecontX and prevent it from completely removing suspect contamination (page 6, line 42):*

*“In some cases, DecontX was not able to completely remove potentially aberrant expression of cell type markers. For example, 43.03% of NK-cells in the PBMC 4K still exhibited expression of some T-cell markers. One potential explanation is that some cells in this dataset were actually NKT-cells and truly share expression features from both NK- and T-cell populations [19]. Another factor is that for cell populations that share substantial similarities in gene expression patterns, DecontX will tend to be conservative and treat the majority of these counts as native expression rather than contamination. In general, we believe this behavior is desired so true biological variation between cell types is not removed.”*

2. In figure 5A-C, the clustering performance was evaluated before and after decontamination by DecontX, and silhouette width was calculated as a quantitative evaluation. In figure 5C, the authors only claimed that the mean silhouette width was increased from 0.04 to 0.06 after decontamination. To present the difference of silhouette width between before and after decontamination, it is better to show the histogram distribution of silhouette.

*We have added the distribution of silhouette width for each cluster to show the improvement of cluster stability using decontaminated counts in Supplementary Figure 6C.*

Reviewer #2:

1. The authors should introduce other algorithms of doublet removers such as scds (10.1093/bioinformatics/btz698) and Scrublet (10.1016/j.cels.2018.11.005) in the section of Introduction for the reader's understanding. The description would highlight the originality of this paper.

*Thank you for the suggestion. We have included the description of doublet problem and current computational methods to identify doublets in the Introduction (page 2, line 8):*

*“Another commonly occurred problem in scRNA-seq methods is when more than one cell is captured in a droplet, also known as a “doublet” or “multiplet”, In microfluidic systems, the occurrence of doublets is proportional to the concentration of cells in the suspension and capture rate of the device [ 10, 11, 12, 13]. Several computational methods have been developed to detect doublets for scRNA-seq data. Scrublet [10] and DoubletFinder [11] simulate artificial doublets from the original data coordinates in a reduced-dimensional representation, then create doublet score for each barcode by calculating the similarity of its representation with artificial doublets. Other approaches such as demuxlet [12] and scds [13] model gene expression from the original data, then assign doublet to barcodes that have observed expression from genes that are likely to not occur simultaneously. While it is important to identify doublets in scRNA-seq, these approaches do not address the problem of contamination caused by ambient RNA or other experimental factors.”*

2. In this paper, the authors developed DecontX for drop-based scRNA-seq. However, in fact, there was a significant impact on CEL-seq2, which is one of the plate-based protocol. The authors more deeply and carefully should discuss the reason for the high contaminant in CEL-seq2. CEL-seq2 might use cell sorter. In general, ambient RNA cannot be contaminated in each cell when use cell sorter. The author expected the steps of library preparation is critical. Which step of library preparation do you think has the problem? Cell sorting, doublet, cell debris, contamination from aerosol, abnormal multiplexing procedure, contamination of genome sequence, or cell barcode swapping? Some papers pointed out that CEL-seq2 shows the irregular distribution of the genomic mapping region. Many of CEL-seq2's reads abnormally are map the intergenic and intron region (Fig. 1. in Elisabetta Mereu et al. bioRxiv).

As the reviewer notes, contamination can occur via mechanisms other than ambient RNA. Unfortunately, we cannot definitively pinpoint the exact step in the CEL-seq2 experimental procedures that cause excess contamination with our algorithm given that we only examine the “down-stream” data at the end of the protocol. We believe that it is not likely due to the sorting procedures or doublets. In this dataset, it is also not likely to be cell barcode swapping during sequencing as that is a specific phenomenon on the flow cells with Exclusion Amplification chemistry (HiSeqX, HiSeq4000, and NovaSeq) [7] and the CEL-seq2 datasets we analyzed were sequenced on the Nextseq 500 [8]. Another possible mechanism of cross contamination in plate-based protocols may occur from evaporation in the wells. The SORT-seq protocol uses vapor-lock oil to prevent evaporation and had less contamination than CEL-seq2 despite being a similar protocol overall.

*We have added text to the introduction and discussion to describe the idea that contamination can happen from ways other than ambient RNA. Specifically, in the background, we add (page 1, line 62):*

*“Beyond ambient RNA, other technical factors may cause contamination between cells such as evaporation in plate-based protocols [7, 8] or barcode swapping during sequencing [9].”*

*In the discussion (page 6, line 56), we note:*

*“Additionally, DecontX estimation on benchmark datasets shows 10x Chromium has lowest contamination, while CEL-seq2 has highest contamination. CEL-seq2 has shown much higher frequencies of intronic and intergenic mappings compared to other scRNA-seq methods [20]. Although the SORT-seq protocol is similar to CEL-seq2, it used vapor-lock oil to prevent evaporation [7] which resulted in less estimated contamination overall in our analysis.”*

3. In the manuscript, the contamination rate is estimated in several protocols. However, the contamination rate is affected by experimental environments such as tissue dissociation protocol and the skill of a person. The authors should investigate the variation of contamination rate among difference data by the same protocol if you claim the difference of contamination rate between each protocol.

*Thank you for suggesting this. We have conducted contamination analysis on 6 additional datasets from three different tissues profiled using two 10X protocols. The analysis is now added to the result (page 5, line 36):*

*“To investigate the variation of contamination levels between cell types from different tissues and 10X protocols, we applied DecontX to six additional datasets including brain cells, heart cells from an E18 mouse profiled using 10X V2 and V3 chemistries (BrainV2 and BrainV3, HeartV2 and HeartV3) as well as PBMCs from a healthy human donor (PBMCV2 and PBMCV3). All six datasets each had between 712 and 1301 cells detected. The median contamination levels were 1.43% in PBMCV2, 0.56% in PBMCV3, 0.34% in BrainV2, 0.03% in BrainV3, 0.23% in HeartV2, and 0.12% in HeartV3 (Figure 6D, Supplementary Figure 8). Therefore, the V3 datasets had lower contamination levels than V2 datasets, indicating that improvement in experimental protocols can decrease overall contamination levels. Furthermore, PBMCs exhibited over 2-fold higher levels of contamination compared to the brain and heart datasets suggesting that contamination levels may also depend on cell types being examined. Overall these results demonstrate that DecontX can measure contamination from ambient RNA or other sources in the library preparation process and will be a useful method to assess the quality of different experimental protocols.”*

4. If the graphical model of the statistical model is shown, it will help the reader understand.

*We have added the graphical model in the Methods section to facilitate understanding of the statistical method (page 8).*

5. In figure 2, Why is the proportion of contamination in the human cell less than that of the mouse cell?

*This is an import point and worth discussing. Cell types with higher total mRNA abundance (i.e. more UMI counts) and that are more prevalent within the dataset are more likely to contribute contaminating reads in other cell types. To emphasize this idea, we have added the following text to the discussion (page 6, line 26):*

*“We observed that cell types with higher total mRNA abundance and prevalence within the dataset are more likely to contribute to the contamination in other cell types. For example, in the human-mouse mixture dataset, the human cells had more uniquely aligning UMIs on average than mouse cells. Therefore, the human cells contributed more reads to the ambient RNA and resulted in more contamination in mouse cells.”*

6. In figure 4, why is the mixture of monocyte the majority? Could you discuss the biological or technical reasons?

*Similar to the previous point, prevalent cell types with higher UMI counts will contribute more to the ambient RNA and thus the contamination in other cell types. In this case, monocytes were the 2nd most common cell type behind T-cells but had higher UMI counts on average than T-cells. Besides the high median level of total UMI counts of monocytes, monocyte-associated marker genes were the most highly expressed cell-type specific markers.*

*We have added the following statement to the result (page 4, line 25):*

*“We also observed that monocytes marker genes were the most prevalent contamination in other cell types (Figure 4). Monocytes had the highest median value of total number of UMIs among the four major cell types (Supplementary Figure 6A) and the monocyte specific markers LYZ, S100A8 and S100A9 had the highest expression levels than other cell type specific markers (Supplementary Figure 6B). Therefore, monocytes marker genes contributed higher probabilities to the contamination distributions in other cell types compared to marker genes from other cell types.”*

*We have also added the following statement to the discussion (page 6, line 32):*

*“In the 4K PBMC data, the monocytes were the second most common cell type and had the highest total numbers of UMI counts on average. In addition, monocyte-associated genes such as LYZ, S100A8 and S100A9 were the most highly expressed cell type specific marker genes and thus contributed more to the contamination in other cell types compared to markers from other populations. These results show that contamination distributions in each cell will be dependent on the other cell types in the assay as well as the level of expression of specific genes in those cell types.”*

7. In figure 5, the author should show the names of the cell type in each cluster.

*We have added labels for the major cell types observed in the PBMC data to Figure 5.*

8. Could you more deeply discuss the relationship between highly contaminated genes, gene function, expression level, and cell-type specificity of expression?

*We have added a section to the discussion that clarifies the behavior of the model in different circumstances (page 5, line 61):*

*“In our model, each cell is treated as a mixture of multinomial distributions over genes, one from its native cell population and another from contamination. For each cell population, the contamination distribution is defined as a combination of gene counts from other cell populations. Genes that are more highly expressed (i.e. have more UMI counts) in other cell populations will be more likely to contribute to contamination in the current cell population. Therefore, these genes will have relatively higher probabilities in the contamination distribution compared to the expression distribution in native cell population and counts for these genes will be more likely to be called “contamination”. Certain types of housekeeping genes, such as ribosomal protein coding genes, can be highly expressed across many cell populations and thus will have high probabilities appearing in both the native cell populations and contamination distributions. In this case, counts for these genes will predominantly be called “native” assuming the overall proportion of estimated contamination in that cell is also relatively low.”*

**Second round of review**

**Reviewer 2**

This revised manuscript is well organized and fully addressed to all concerns.
